# DDR2‐mediated autophagy inhibition contributes to angiotensin II‐induced adventitial remodeling

**DOI:** 10.1002/ctm2.70361

**Published:** 2025-06-04

**Authors:** Gaojian Huang, Zhilei Cong, Yuhao Zhao, Tong Zhu, Ruosen Yuan, Zhen Li, Xuelian Wang, Jia Qi

**Affiliations:** ^1^ Department of Clinical Pharmacy Xinhua Hospital affiliated to Shanghai Jiaotong University School of Medicine Shanghai China; ^2^ Department of Cardiology Shanghai Ninth People's Hospital affiliated to Shanghai Jiaotong University School of Medicine Shanghai China; ^3^ Department of Emergency Huashan Hospital affiliated to Fudan University Shanghai China; ^4^ Department of Geriatric Xinhua Hospital affiliated to Shanghai Jiaotong University School of Medicine Shanghai China; ^5^ Department of Cardiology Ruijin Hospital affiliated to Shanghai Jiaotong University School of Medicine Shanghai China

**Keywords:** adventitial fibroblasts, autophagy, discoidin domain receptor 2, phenotypic switch

## Abstract

**Aims:**

Adventitial remodelling in hypertension is characterized by a transformation of adventitial fibroblasts (AFs) into myofibroblasts. Previous studies have highlighted the crucial role of discoidin domain receptor 2 (DDR2) in vascular remodelling. Since DDR2‐sustained tyrosine phosphorylation activates PI3K, which may inhibit autophagy through the mTOR signalling pathway, we aimed to investigate whether DDR2 contributes to mTOR‐mediated autophagy suppression and subsequently promotes AFs transformation and adventitial remodelling.

**Methods and results:**

Single‐cell RNA sequencing revealed that DDR2 was upregulated in adventitial fibroblasts (AFs) in angiotensin II (Ang II, 1000 ng/min/kg) administrated wild‐type (WT) mice. In AFs, rapamycin, an autophagy agonist, significantly attenuated Ang II‐induced autophagy suppression and phenotype switching, whereas the autophagy inhibitor chloroquine (CQ) exacerbated these effects. DDR2 inhibition significantly alleviated PI3K/Akt/mTOR pathway‐mediated autophagy suppression and subsequently inhibited AFs phenotypic switching. Conversely, DDR2 overexpression aggravated autophagy suppression and AFs phenotypic switching. Consistent with the cellular findings, prophylactic administration of rapamycin (4 mg/kg/d) or conditional knockout of *Ddr2* in mice ameliorated autophagy suppression, AFs differentiation and adventitial remodelling in vivo.

**Conclusion:**

DDR2 serves as a critical mediator of autophagy suppression during Ang II‐induced phenotypic transformation of AFs and adventitial remodelling. Targeting DDR2 signalling attenuates autophagy dysfunction and inhibits AFs activation, thereby mitigating pathological adventitial remodelling. These findings highlight DDR2 as a potential therapeutic target for preventing conditions driven by aberrant adventitial remodelling.

## Graphical abstract

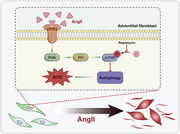

## 1 INTRODUCTION

Vascular remodelling is a critical factor in the aetiology and progression of numerous cardiovascular disorders, such as hypertension. The structure of the blood vessel wall encompasses three distinct layers: the intima, which is the innermost layer, the media in the middle, and the adventitia, which is the outermost layer. Although intima and media have gained more attention, adventitia, mainly composed of adventitial fibroblasts (AFs), also has been recently found to act as a key regulator of vascular wall structure and function. Adventitial remodelling is featured with the thickening of the adventitial layer, the increased proliferation of AFs, and phenotypic differentiation of fibroblasts to myofibroblasts, which presents with the vast expression of α‐smooth muscle actin (α‐SMA) and collagen I1. Over the past years, the outside‐in hypothesis has emerged, whereby vascular remodelling is initiated in the adventitia. Of note, angiotensin II (Ang II) has been shown to activate multiple signalling pathways in vascular cells including AFs, vascular smooth muscle cells and endothelial cells, which are critically involved in adventitial remodelling, elastin degradation and aneurysms/dissections formation.^2^, ^3^, ^4^ Although emerging evidence, including our early research, supports that excessive adventitial remodelling aggravates vascular alteration in Ang II–induced hypertension,^5^, ^6^, ^7^ more underlying mechanisms and molecular targets are still needed to be investigated pressingly.

Discoidin domain receptor 2 (DDR2) belongs to the family of the DDR comprising two distinct members (DDR1,2) that are characterized as receptor tyrosine kinases (RTKs) and are expressed predominantly in cells of mesenchymal origin such as fibroblasts.^8^ It has been diffusely reported that DDR2 regulates collagen expression and cellular processes such as cell proliferation, migration, and differentiation in a variety of malignancies.^9^, ^10^ Moreover recent studies advocated that abolition of DDR2 reduces lung fibrosis and angiotensin‐induced cardiac fibrosis.^10^, ^11^ Furthermore, it is well known that phosphatidylinositol 3‐phosphate kinase (PI3K)/Akt/mTOR pathway regulates autophagy in a wide range of cell types and was also found to be significantly activated in hypoxia‐induced AFs proliferation.^12^, ^13^ Interestingly, perhaps one role of DDR‐sustained tyrosine phosphorylation is involved in the activation of a series of signalling proteins such as PI3K. Although these observations support that broadly expressed DDR2 may mediate autophagy and vascular remodelling, it remains elusive that whether DDR2 in AFs promotes mTOR‐mediated autophagy inhibition and contributes to adventitial remodelling in hypertensive mice through.

Herein, we show that DDR2 deficiency in fibroblast significantly attenuates Ang II‐induced phenotypic switching of AFs and adventitial remodelling, and vice versa. Mechanistically, the DDR2 deletion blocks PI3K/Akt/mTOR mediated autophagy inhibition, which subsequently diminishes ROS production.

## 2 METHODS

### 2.1 Antibodies, Lentiviruses and Chemicals

Collagenase type II was purchased from Sigma. Antibodies against SQSTM1(#5114), ATG7 (#8858), ATG5(#12994), p‐ULK1(Ser757) (#14202), 4EBP‐1 (#9644T), LC3B (#83506), GAPDH (#5174), α‐SMA (#19245), p‐AKT (#9018), AKT (#4691), mTOR(#2972), p‐mTOR(#2971) and Collagen I (#72026) were obtained from Cell Signaling Technology. ULK1 (#A8529), p70 S6 Kinase (#A2190), Phosoho‐p70 S6 Kinase (#AP0502), P‐4EBP‐1(#AP 0032), PIK3CA (#A0265) were obtained from Abclone. Anti‐DDR2 antibody (#ab126773) was obtained from Abcam. Western Secondary antibodies (Peroxidase AffiniPure Goat Anti‐Rabbit IgG, #111‐35003; Peroxidase AffiniPure Goat Anti‐Mouse IgG, #115035003), Alexa Fluor 594‐labelled anti‐rabbit IgG (#711585152), Alexa Fluor 488‐labelled anti‐rabbit IgG (#715545150) were supplied by Jackson immune‐research. DDR2 shrna lentivirus and DDR2‐overexpressing lentiviruses were synthesized by HanbioBiotech Co., Ltd. rapamycin (#HY‐10219), Chloroquine diphosphate (#HY‐135811A), N‐acetylcysteine (NAC, #HY‐B0215), WRG‐28 (#HY‐114169), losartan (#HY‐17512), PD 123319 (HY‐10259A) and LY294002 (#HY‐10108) were obtained from Medchemexpress (USA).

### 2.2 Animal models and treatments

DDR2^flox/flox^ (DDR2^fl/fl^) mice with loxP sites flanking exons 9 of *Ddr2* gene were generated by Shanghai Model Organisms (#NM‐CKO‐200311). S100a4‐CreERT2 (FSP1‐Cre) mice were a gift from GemPharmatech (#T006589). Fibroblast‐specific DDR2 knockout mice (DDR2‐cKO) were created by crossing DDR2^fl/fl^ mice with S100a4‐CreERT2 transgenic mice.^14^ To induce Cre‐mediated recombination, 8‐week‐old male DDR2‐cKO mice received intraperitoneal injections of tamoxifen (75 mg/kg/day, dissolved in corn oil; Sigma‐Aldrich, #T5648) for 5 consecutive days.^15^ Animal protocols were approved by Animal Research Committee of Xinhua Hospital, Shanghai Jiaotong University School of Medicine and daily health monitoring confirmed no adverse events or fatalities during the study. Littermate DDR2^fl/fl^ mice (Cre‐negative) were used as controls to ensure genetic and environmental consistency. The Adventitial remodelling model was established as described previously.^3^, ^16^ Briefly, male wild‐type (WT), DDR2^fl/fl^, and DDR2‐cKO mice (8‐week‐old) were administrated Ang II (1000 ng/min/kg, Abcam, ab120183) or saline via subcutaneous infusion for 4 weeks through Alzet miniosmotic pumps, while the each group of WT was further divided into two groups (subcutaneous injection with PBS or rapamycin(4 mg/kg/d)).

### 2.3 Aortic dissociation and single cell preparation

After the adventitial remodelling model was established, mice were euthanized by CO_2_ overdose and aorta was harvested. Aortas from five mice in each group (control and Ang II) were pooled, longitudinally opened, and the adventitia were stripped for RNA sequencing. Single‐cell suspension of adventitial cells was prepared using an enzymatic digestion protocol previously described.^17^ In brief, after rinsing with 1% PBS, the adventitia was cut and digested with an enzyme digestion [2 mg/mL collagenase type I (Invitrogen, #17018‐029), 2 mg/mL disapase II (Sigma, #C2‐BIOC) in Hank's balance salt solution containing calcium and magnesium] for 2 h at 37°C. The cell suspension was filtered through a 70 µm mesh, washed twice in the 1 × PBS, and resuspended at 1 × 10^6^ cells per ml in 1 × PBS containing 0.04% bovine serum albumin, before being subjected to scRNA‐seq.

### 2.4 Single‐Cell RNA Sequencing

The scRNA‐seq was conducted using the Standard 10× Chromium Single Cell 3′ v3 protocol by 10X Genomics GemCode Technology. In summary, single cells were individually tagged with a unique 10× Barcode and unique molecular identifier through the encapsulation into Gel Bead‐In‐Emulsions. Sequences sharing identical 10× Barcodes were then classified as originating from the same cell. The resultant cDNA library was prepared and subjected to sequencing on the Nova PE150 platform.^17^

Raw reads were demultiplexed and mapped to the reference genome using the 10X Genomics Cell Ranger pipeline (https://www.10xgenomics.com/support) with default parameters. The Cell Ranger mkfastq command was employed to generate Fastq files, and the data were then mapped to the pre‐built mouse reference genome (mm10, version 1.2.0).^18^

After aggregating samples from aortic adventitial cells, the Seurat v3.0 software package in R was deployed to carry out a series of data processing steps. These included filtering of cells, data normalization, conducting principal component analysis, detecting variable genes, forming clusters, and generating a t‐SNE plot for visualization purposes. Gene expression was visualized using Seurat functions VlnPlot, FeaturePlot and DotPlot for violin plots, feature plots, and dot plots, respectively. Specific cluster markers were identified using the Seurat function FindAllMarkers. Data generated in present study will be made available in the Gene Expression Omnibus repository upon acceptance.

### 2.5 Histological and immunofluorescence, transmission electron microscopy

The sample preparation and analysis of the adventitia were performed following previously established methods.^5^ Briefly, thoracic aortas were collected and fixed in 4% paraformaldehyde for 24 h. After being embedded in paraffin, 5‐µm transverse histological sections were cut and stained with haematoxylin‐eosin (H&E), or used for additional special staining and immunostaining. The adventitial thickness and fibrosis area, primarily in the ascending aorta, were quantified using Image J software. Additionally, adventitial samples from the mice were fixed in 2.5% (v/v) glutaraldehyde. Ultrathin sections were prepared and examined using a Philips CM 10 transmission electron microscope.

### 2.6 Primary AFs cell culture, cell proliferation and migration assays

The methodology for the extraction and characterization of AFs from the healthy thoracic aortas of male C57BL/6J mice has been previously documented in our earlier published study.^5^ The EdU assay was employed to assess cell proliferation, the proliferation rate was determined by calculating the percentage of EdU‐positive cells relative to the total cell count.^19^ The scratch‐wound assay and transwell assay were used to evaluate the capabilities of cell migration.^5^, ^19^ AFs were transfected with lentivirus for 24 h or pretreated for 1 h with pharmacological agents (LY294002 NAC, rapamycin, CQ). For the scratch‐wound assay, a standardized scratch was generated in confluent monolayers using a p200 pipette tip. Cells were maintained in serum‐free medium for 24 h at 37°C, and wound closure was quantified as: Migration area (%) = *(A_0_ ‐ A_n_)/ A_0_* × 100, where *A_0_* and *A_n_* represent the initial and residual wound areas. For the transwell assay, 2 × 10⁴ cells in serum‐free medium were seeded into 8.0 µm Transwell inserts (Corning, NY, USA).Inhibitors or Ang II were added to the lower chamber and migrated cells were fixed with methanol after 24 h, stained with Crystal Violet (China) and counted in five random fields per membrane under a phase‐contrast microscope (Leica).

### 2.7 Western blot analysis

Tissues from mice and treated or control AFs were collected, washed and lysed as previously reported.^5^ Equal loading of protein was assessed by BCA, and polyvinylidene difluoride membrane was used for transfers, which was blocked with 5% BSA. The membranes were incubated with primary antibodies against a range of proteins including Collagen I, α‐SMA, GAPDH, DDR2, p‐AKT, AKT, p‐mTOR, mTOR, p‐p70S6K, t‐p70S6K, p‐4EBP‐1, t‐4EBP‐1, p‐ULK1, t‐ULK1, ATG7, ATG5, LC3B, and SQSTM1. All primary antibodies were diluted to 1:1000 using dilution buffer from Beyotime. Subsequently, the membranes were exposed to corresponding secondary antibodies. After thorough washing, protein signals were captured by the Enhanced chemiluminescence Western blot detection system and the bands were quantified by Image J software.

### 2.8 Co‐immunoprecipitation

Co‐immunoprecipitation was performed using a Pierce™ Co‐Immunoprecipitation Kit (Thermo Fisher, #26149) to assess the interaction between DDR2 and PI3K, following the manufacturer's instructions. Briefly, affinity‐purified antibodies against PI3K (25 µg) or DDR2 (25 µg) were immobilized onto the resin for 90 minutes at room temperature. After three washes with 1× coupling buffer, 500 µg of protein lysate was added to the resin, and the mixture was incubated overnight at 4°C. After incubation, the resin was washed four times before the immunoprecipitated proteins eluted and analyzed by Western blotting.

### 2.9 Reactive oxygen species measurement

For the quantification of intracellular reactive oxygen species (ROS), cells were stained using MitoSOX (5 µM, Thermo Fisher, #M36009) for a duration of 10 min prior to being fixed with 4% paraformaldehyde. Subsequently, coverslips were mounted, and the cells were examined using a confocal fluorescence microscope. Image J software quantified Fluorescence intensity across 4–5 separate fields.

### 2.10 PI3K activity assay

PI3K activity in cell lysates was measured utilizing the PI3K activity assay kit (Echelon Biosciences, #K‐1000s) as per the guidelines provided by the manufacturer. These experiments were conducted in triplicates, and the outcomes from treatments were compared with the baseline values from control samples.

### 2.11 Transfection

Lentivirus harbouring DDR2 RNA interference (LV‐sh‐DDR2) and DDR2 over‐expression (LV‐DDR2) were designed by HanbioBiotech Co., Ltd. AFs underwent viral infection at a multiplicity of infection (MOI) of 50. Prior to initiating the main experiments, the efficacy of the infection was verified using Western blot analysis. The AFs were infected by dual‐tagged LC3 (mRFP‐GFP‐LC3) lentivirus (Shanghai Genechem Co.,Ltd.) for autophagy flux analysis. Briefly, the cells were infected by lentivirus (20 MOI) for 4 h followed by a treatment with Ang II (100 nM) and rapamycin (1 µM) for 24 h. Following fixation using 4% paraformaldehyde, cells were examined under a fluorescence microscope, and the quantities of GFP and mRFP dots were ascertained through manual enumeration of fluorescent puncta across 10 separate fields derived from three distinct fibroblast preparations. The ratio of dots per cell was quantified by dividing the aggregate number of dots by the total count of nuclei within each individual microscopic field.

### 2.12 Statistical analysis

Statistical evaluations were conducted using Graph‐Pad Prism software (version 10.4). All experiments were performed with a minimum of three independent biological replicates. Data are expressed as mean ± standard error of mean (SEM). Comparisons among multiple groups were analyzed using two‐way analysis of variance (ANOVA), followed by Bonferroni's post‐hoc test for multiple comparisons. Non‐parametric Kruskal–Wallis tests with Dunn–Benjamini–Hochberg post‐hoc corrections were applied for multi‐group comparisons of protein levels. Differences between two groups were compared by Student's *t‐*test (or Mann–Whitney U test for non‐parametric data). A *p*‐value less than 0.05 was deemed to denote statistical significance.

## 3 RESULTS

### 3.1 DDR2 is highly expressed in adventitial fibroblasts and upregulated in Ang II induced hypertensive mice

**FIGURE 1:**
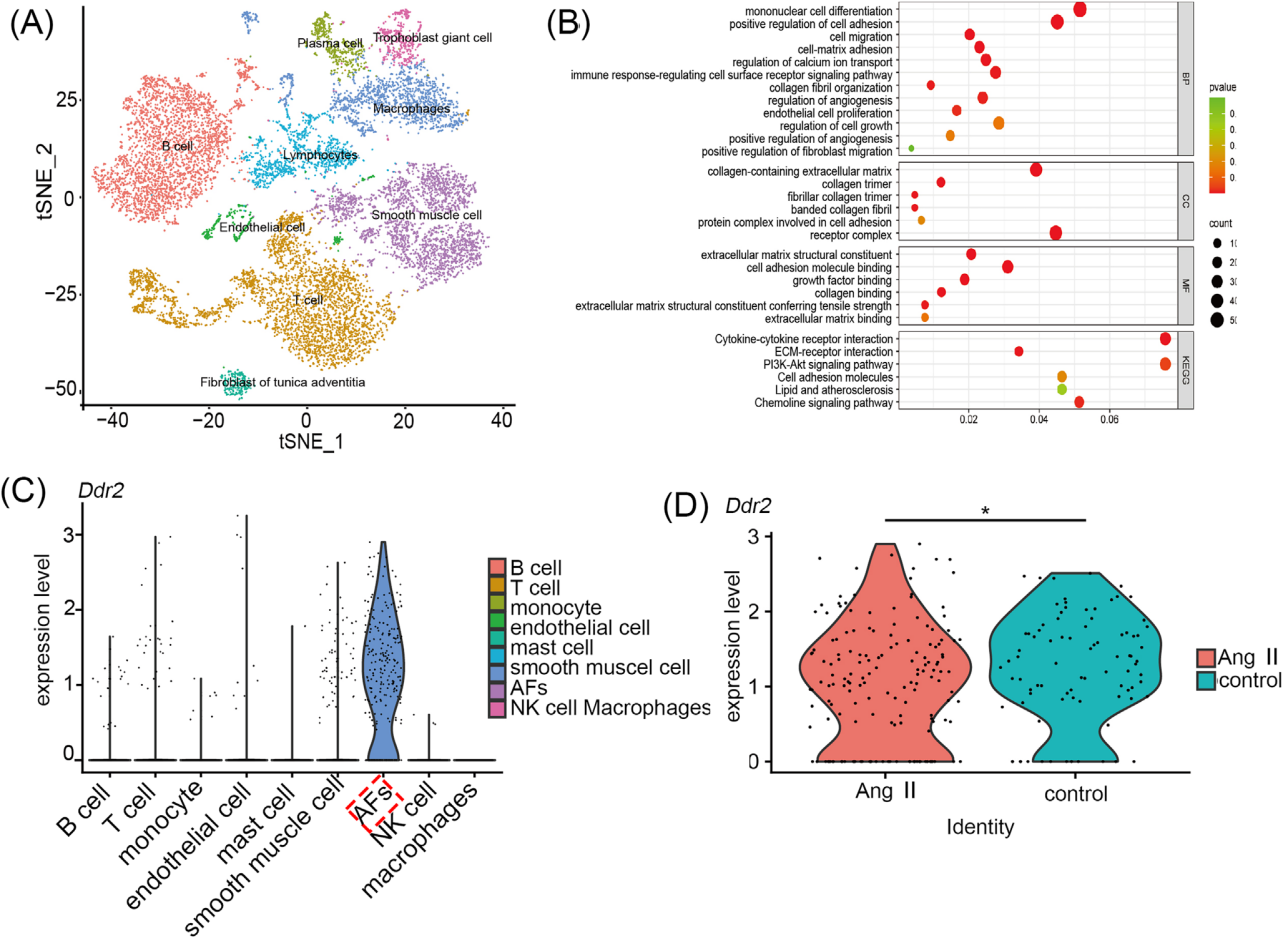
Single‐cell analysis of adventitial cells and discoidin domain receptor 2 (DDR2) expression following Ang II stimulation. (A) t‐distributed stochastic neighbour embedding (t‐SNE) plot of adventitial cells, with colours representing different cell clusters. Cluster 1: B cells; Cluster 2: T cells; Cluster 3: Plasma cells; Cluster 4: Endothelial cells; Cluster 5: Fibroblasts of the tunica adventitia; Cluster 6: Lymphocytes; Cluster 7: Macrophages; Cluster 8: Smooth muscle cells; Cluster 9: Trophoblast giant cells. (B) Selected top Gene Ontology (GO) terms of adventitia cells after Ang II treatment. (C) Violin plot of Ddr2 gene expression across cell clusters. (D) Violin plot showing the expression of selected Ddr2 markers following Ang II stimulation, compared with the control group. Statistical significance was assessed using unpaired Student's t‐test. *p < 0.05.

To characterize the adventitial cellular landscape, we isolated and enzymatically dissociated adventitial cells from male WT mice 12 weeks of age for scRNA‐seq. After quality control, a total of 6332 cells from the control group and 6058 cells from the Ang II‐infused group were selected for further analysis. Unbiased clustering using Seurat canonical correlation analysis revealed 9 clusters, which were visualized with t‐distributed stochastic neighbour embedding (Figure 1A). Most cell types were identified, including B cells, T cells, NK cells, Endothelial cells, AFs, smooth muscle cells, Mast cells and Macrophages, with matched transcriptional profiles and putative biological roles (Figure 1A). To decipher the functional differences after Ang II stimulation, we used gene ontology (GO) analysis to examine changes in gene molecular functions, cellular components, and biological processes. GO analysis indicated that after Ang II stimulation, the main effects were on cell proliferation, migration, extracellular matrix adhesion, collagen fibre organization, and positive regulation of angiogenesis (Figure 1B). Single‐cell transcriptomic analysis revealed constitutive high expression of Ddr2 in AFs, with a unimodal expression distribution (Figure 1C). Comparative analysis demonstrated that Ang II treatment increased the proportion of Ddr2 (Figure 1D). Building upon single‐cell transcriptional evidence of Ddr2 upregulation, our time‐course Western blot analysis (Figure S1) revealed that Ang II induced rapid DDR2 protein accumulation in AFs, with detectable increases as early as 5–10 min post‐stimulation.

### 3.2 DDR2 deficiency restores Ang II‐induced AFs phenotype switching and autophagy inhibition

**FIGURE 2:**
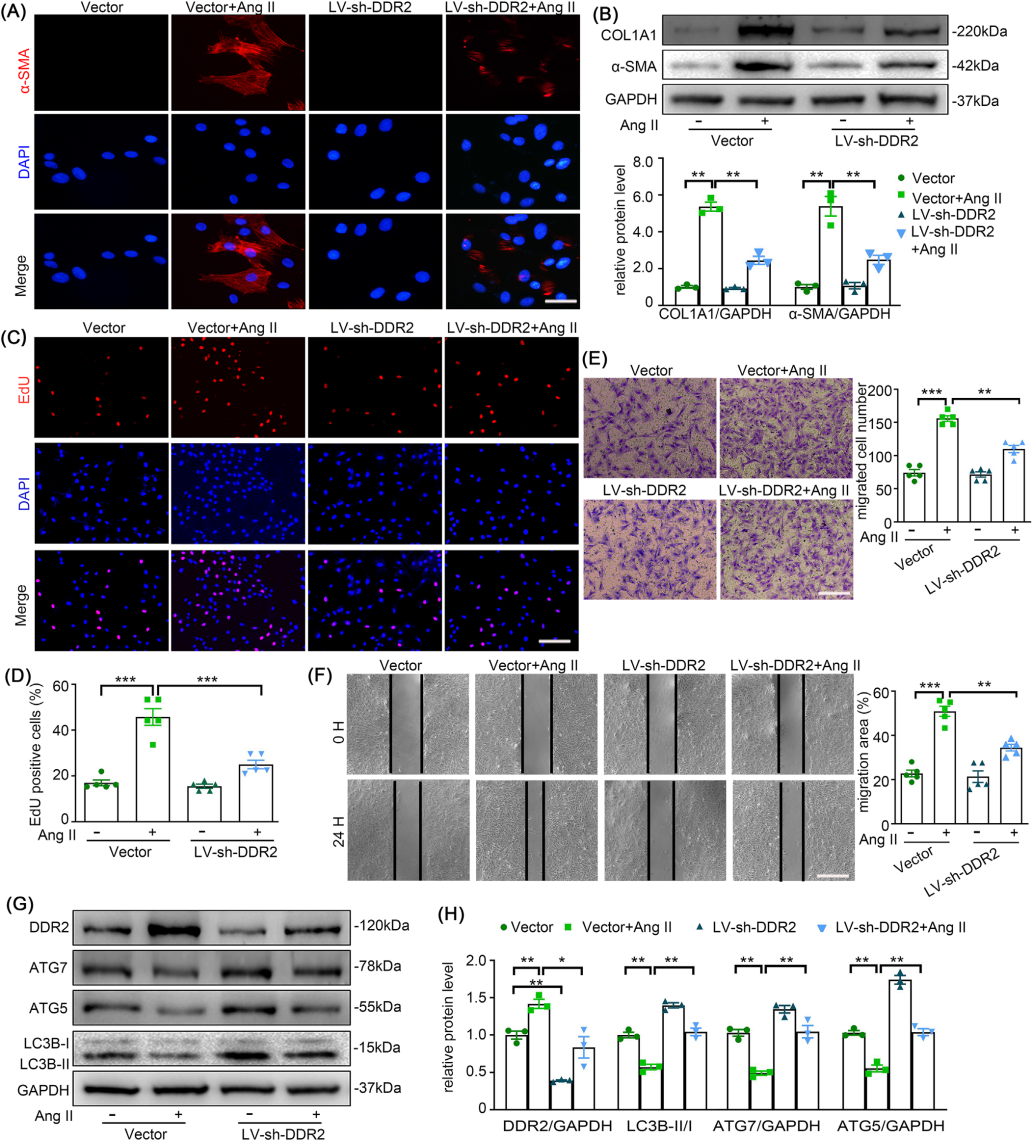
Discoidin domain receptor 2 (DDR2) deficiency relieved Ang II‐induced AF phenotype switching and autophagy inhibition. AFs were infected with LV‐sh‐DDR2 or scramble for 24 h, followed by treatment with Ang II (100 nmol/L) for 24 h. (A) AF phenotype switching was observed by immunofluorescence staining with α‐SMA (red) and DAPI (blue). Scale bar = 40 µm. (B) The protein expression of α‐SMA and COL1A1 were examined by Western blotting and quantified by densitometry. (C‐D) Representative images and quantification of EdU staining. Scale bar = 200 µm. (E) Representative images of transwell migration assay and quantification of the migrated cells. Scale bar = 200 µm. (F) Representative images of scratch‐wound assay and quantification of the migration area were presented. Scale bar = 500 µm. (G‐H) The expression of DDR2, ATG7, ATG5 and LC3B were examined by Western blotting and quantified by densitometry. Statistical significance was assessed using two‐way ANOVA followed by Bonferroni test. All values are presented as means ± SEM, n = 3–5 per group; *p < 0.05, **p < 0.01, ***p < 0.001.

Previous studies suggest that abolition of DDR2 reduces lung fibrosis and angiotensin‐induced cardiac fibrosis by regulating the expression of collagen and vital cellular processes,^11^, ^20^ which also indicate that DDR2 might as well be required for adventitial remodelling. Consistent with our prior findings demonstrating Ang II‐induced adventitial remodelling,^5^ Western blotting and immunofluorescence analyses revealed that Ang II mechanistically upregulated α‐SMA and collagen I expression in AFs. These findings collectively confirm Ang II‐driven phenotypic switching of AFs toward a pro‐fibrotic myofibroblast state.^21^ Present results also showed that Ang II induced acute activation of DDR2 within minutes (Figure S1). To delineate DDR2's functional involvement in Ang II‐induced AFs activation, we systematically assessed phenotype switching, proliferative capacity, and migratory responses through integrated experimental approaches. As shown in the Figure 2A,B, the phenotypic transition of AFs following Ang II stimulation was attenuated by the transfection of LV‐sh‐DDR2 as determined by the lower expressions of α‐SMA and Collagen I in LV‐sh‐DDR2 + Ang II treated cells in contrast to Ang II treated AFs. And as shown in Figure 2C–F, LV‐sh‐DDR2 reduced Ang II‐mediated proliferation and migration of AFs.

As previous studies have shown that autophagy may mediate AFs activation in atherosclerosis,^22^ we also investigated the effect of DDR2 on autophagy. Interestingly, autophagy suppression by Ang II was restored in DDR2 deficient AFs (Figure 2G,H). These results suggest that suppression of DDR2 alleviates Ang II‐induced AFs phenotype switching, and thus there could be a link between DDR2 and autophagy.

### 3.3 DDR2 overexpression accelerates Ang II‐induced autophagy impairment and AFs phenotype switching

**FIGURE 3:**
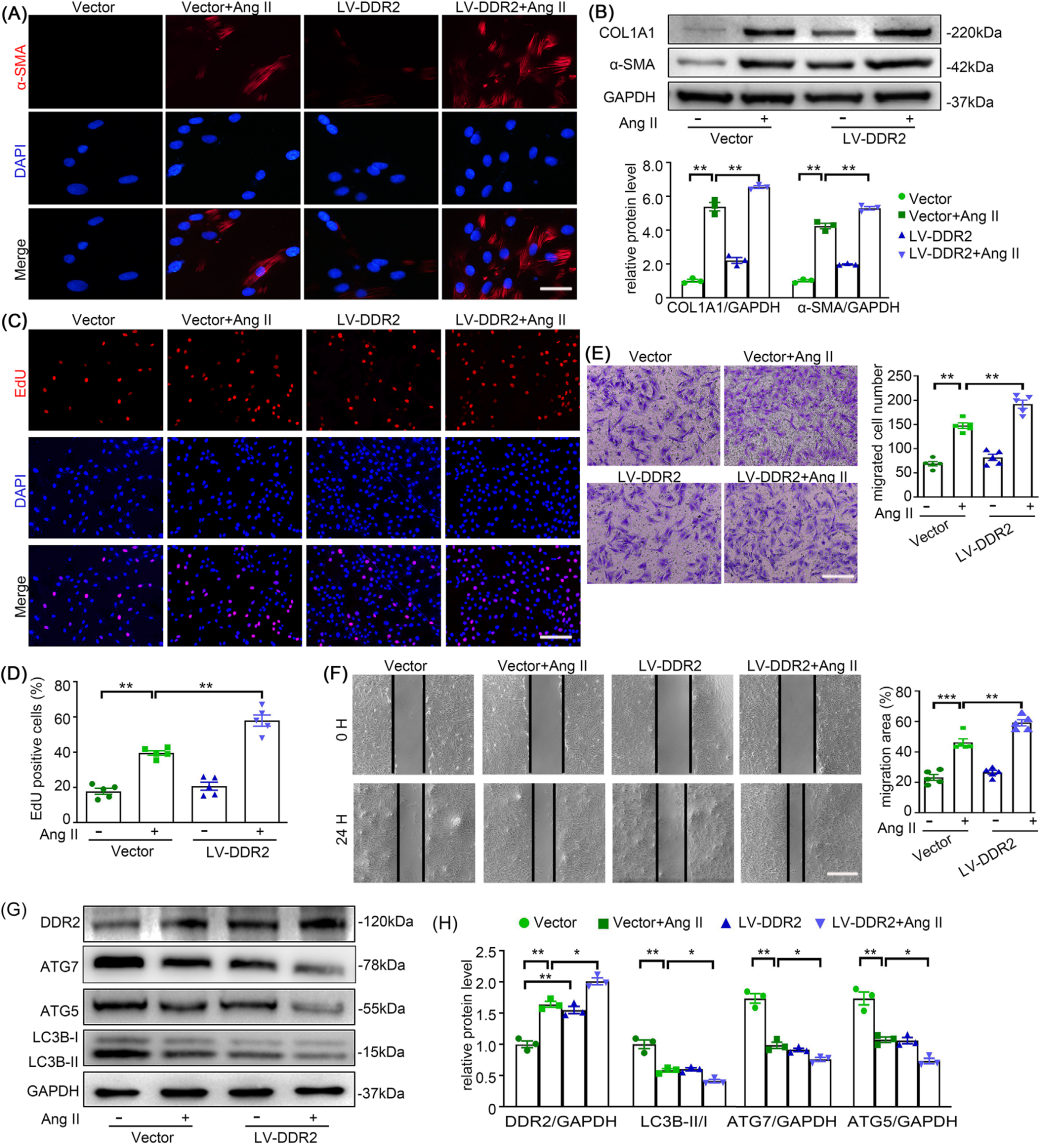
Discoidin domain receptor 2 (DDR2) overexpression accelerated Ang II‐induced autophagy impairment and AF phenotype switching. AFs were infected with DDR2‐overexpressing lentivirus (LV‐DDR2) or vector for 24 h, followed by treatment with Ang II (100 nmol/L) for 24 h. (A) AF phenotype switching was observed by immunofluorescence staining with α‐SMA (red) and DAPI (blue). Scale bar = 40 µm. (B) The protein expression of α‐SMA and COL1A1 were examined by Western blotting and quantified by densitometry. (C‐D) Representative images and quantification of EdU staining. Scale bar = 200 µm. (E) Representative images of transwell migration assay and quantification of the migrated cells. Scale bar = 200 µm. (F) Representative images of scratch‐wound assay and quantification of the migration area were presented. Scale bar = 500 µm. (G‐H) The expression of DDR2, ATG7, ATG5 and LC3B were examined by Western blotting and quantified by densitometry. Two‐way analysis of variance followed by the Bonferroni's post hoc test was used for statistical analyses. All values are presented as means ± SEM, n = 3–5 per group; *p < 0.05, **p < 0.01, ***p < 0.001.

To further explore the role of DDR2 in Ang II treatment, AFs were infected with DDR2‐overexpressing lentivirus (LV‐DDR2). DDR2 overexpression significantly exacerbated the transformation of AFs into myofibroblasts after Ang II administration (Figure 3A,B). In agreement with the phenotypic alterations, the proliferation and migration of AFs were also strengthened by the up regulation of DDR2, after Ang II treatment (Figure 3C,F). Notably, these LV‐DDR2‐induced pro‐fibrotic effects were effectively blocked by the DDR2‐specific inhibitor WRG‐28 (Figure S2). Meanwhile, the combination of Ang II treatment and DDR2 overexpression caused more severe damage to autophagy (Figure 3G,H), implying that DDR2 might mediate autophagy inhibition to promote Ang II‐induced adventitial remodelling.

### 3.4 Rapamycin attenuates Ang II‐induced autophagy inhibition, AFs phenotype switching, proliferation and migration

**FIGURE 4:**
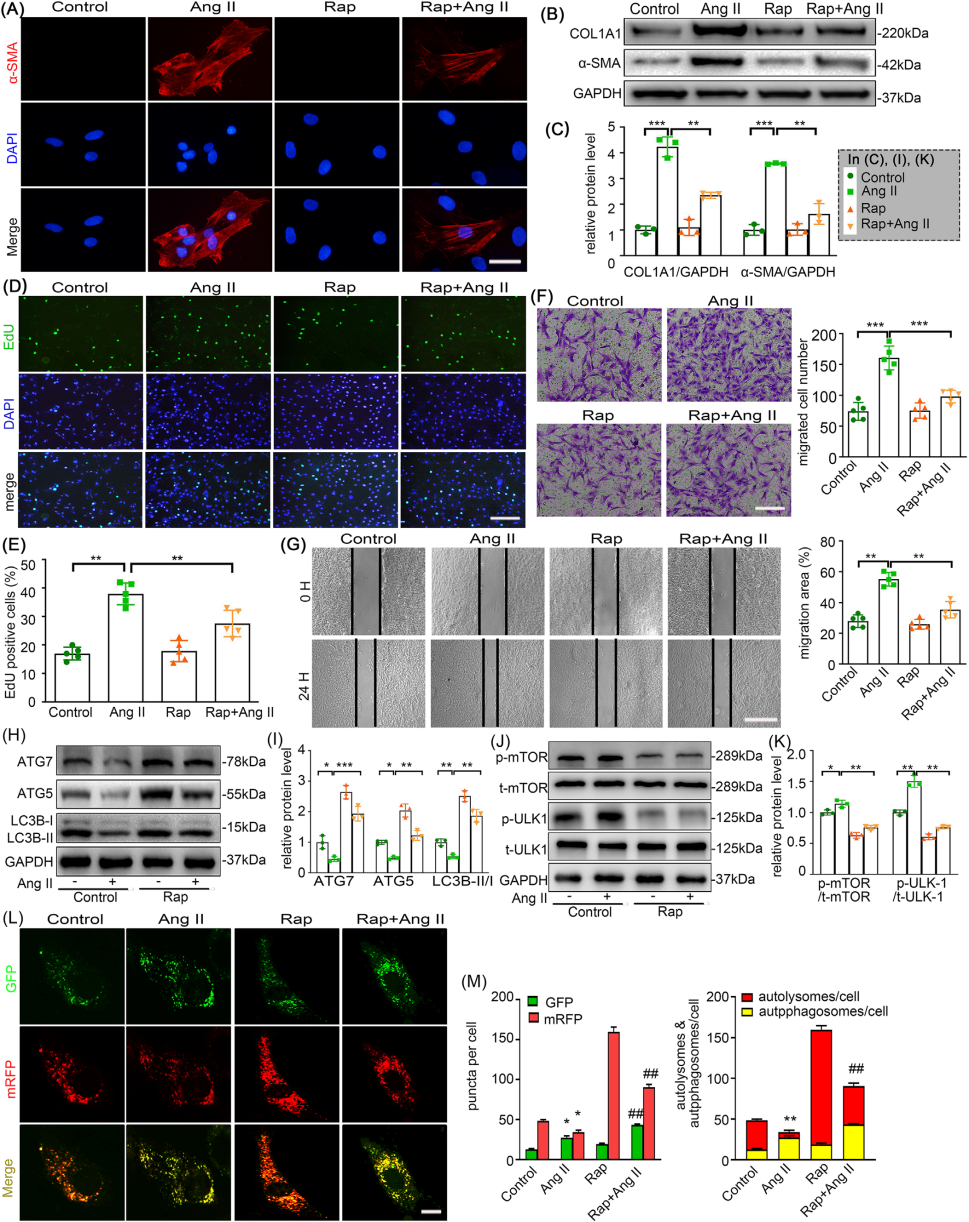
Rapamycin attenuates Ang II‐induced autophagy inhibition, AF phenotype switching, proliferation and migration. Mouse AFs with or without rapamycin (1 µmol/L) pretreatment for 1 h were treated with either the vehicle or Ang II (100 nmol/L) for 24 h.(A) AF phenotype switching was observed by immunofluorescence staining with α‐SMA (red) and DAPI (blue). Scale bar = 40 µm. (B‐C) The protein expression of α‐SMA and COL1A1 were examined by Western blotting and quantified by densitometry. (D‐E) Representative images and quantification of EdU staining. Scale bar = 200 µm. (F) Representative images of transwell migration assay and quantification of the migrated cells. Scale bar = 200 µm. (G) Representative images of scratch‐wound assay and quantification of the migration area were presented. Scale bar = 400 µm. (H‐K) The expression of ATG7, ATG5, LC3B, p‐mTOR, t‐mTOR, p‐ULK1 and t‐ULK1 were examined by Western blotting and quantified by densitometry. (L‐M) AFs were transfected with mRFP‐GFP‐LC3 adenovirus for 12 h, followed by rapamycin and Ang II exposure and assessment via fluorescence microscope. Yellow dots represent autophagosomes, whereas red dots indicate autolysosomes. Scale bar = 10 µm. Statistical significance was assessed using two‐way ANOVA followed by Bonferroni test. All values are presented as means ± SEM, n = 3–5 per group; *p < 0.05, **p < 0.01, ***p < 0.001. Rap indicates rapamycin. GFP indicates green fluorescent protein.

To delineate the inhibitory the effects of rapamycin on Ang II‐mediated AFs activation, cells were pretreated with or without rapamycin (1 µM) for 1 h prior to 24 h stimulation with vehicle or Ang II (100 nM). As demonstrated in Figure 4A,B, rapamycin pretreatment remarkably alleviated Ang II‐induced myofibroblast differentiation, evidenced by reduced α‐SMA and collagen I expression. Following 24 h exposure, Ang II stimulation markedly increased EdU‐positive cells compared to control group, while rapamycin cotreatment substantially suppressed this proliferative response (Figure 4D,E). Consistent with these findings, rapamycin attenuated Ang II‐enhanced AFs migratory capacity, as quantified by transwell (Figure 4F) and scratch assays (Figure 4G). Since Rapamycin stimulates autophagy by inhibiting mTOR signalling, we explored the role of autophagy in Ang II‐stimulated AFs. Western blotting revealed that Ang II downregulated the LC3B‐II/I ratio and expression of ATG5 and ATG7, while enhancing mTOR phosphorylation (p‐mTOR) and inhibiting phosphorylation of ULK1 at Ser757 (p‐ULK1). These effects were attenuated by rapamycin co‐treatment (Figure 4H–K). This indicates that Ang II does impair autophagy in AFs. Additionally, autophagic flux test further confirmed Ang II also inhibits autophagic flux in AFs, whereas rapamycin partially restored it (Figure 4L,M). Collectively, our results propose that rapamycin may play a key role in the inhibition of Ang II‐induced AFs phenotype switching, proliferation and migration via autophagy enhancement.

### 3.5 Chloroquine (CQ) aggravates Ang II‐induced autophagy inhibition and AFs phenotype switching

**FIGURE 5:**
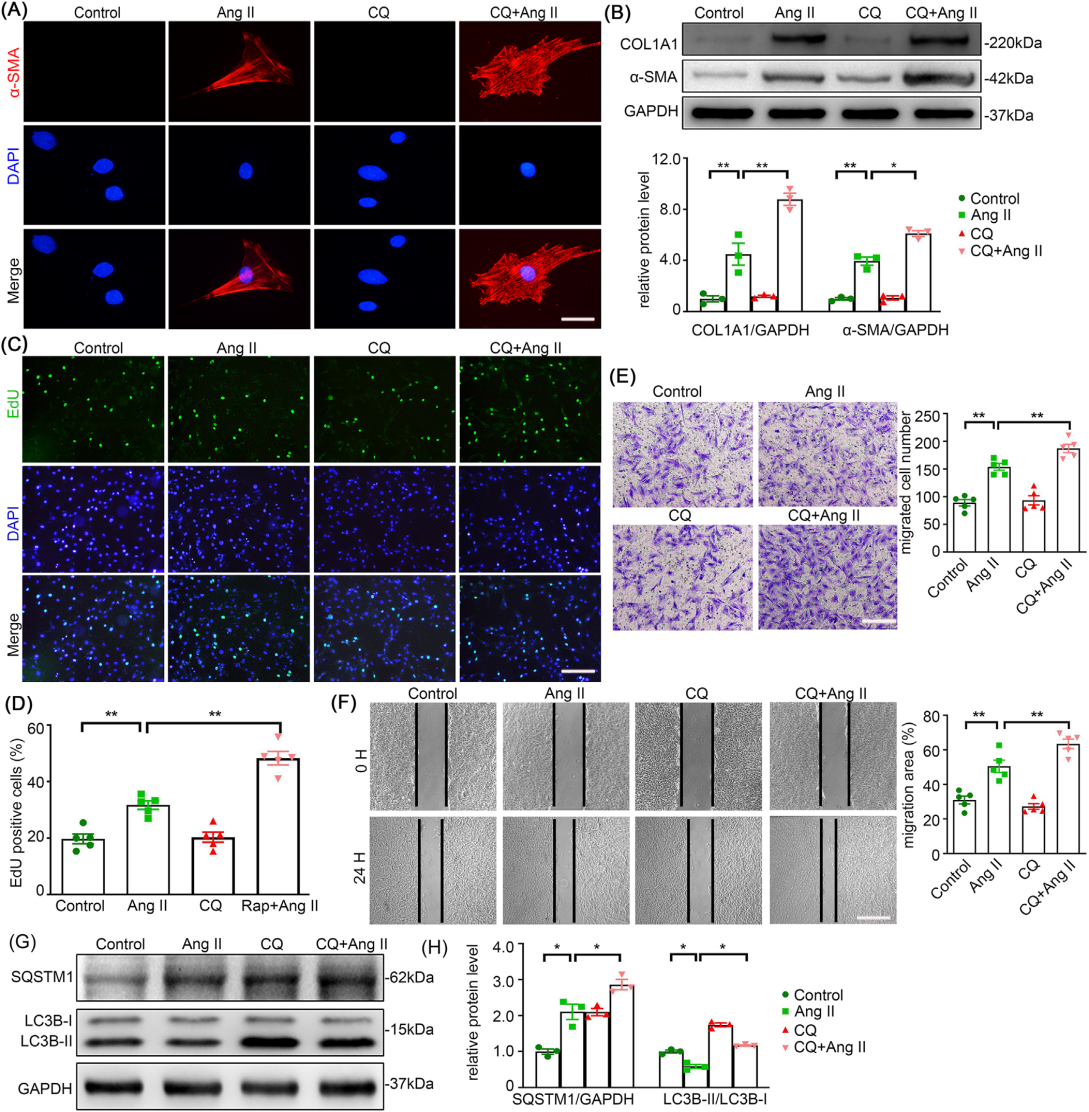
CQ aggravated Ang II‐induced autophagy inhibition and AF phenotype switching. Mouse AFs with or without CQ (20 µmol/L) pretreatment for 1 h were treated with either the vehicle or Ang II (100 nmol/L) for 24 h. (A) AF phenotype switching was observed by immunofluorescence staining with α‐SMA (red) and DAPI (blue). Scale bar = 40 µm. (B) The protein expression of α‐SMA and COL1A1 were examined by Western blotting and quantified by densitometry. (C‐D) Representative images and quantification of EdU staining. Scale bar = 200 µm. (E) Representative images of transwell migration assay and quantification of the migrated cells. Scale bar = 200 µm. (F) Representative images of scratch‐wound assay and quantification of the migration area were presented. Scale bar = 500 µm. (G‐H) The expression of SQSTM1 and LC3B were examined by Western blotting and quantified by densitometry. Two‐way analysis of variance followed by the Bonferroni's post hoc test was used for statistical analyses. Non‐parametric Kruskal‐Wallis tests with Dunn‐Benjamini‐Hochberg post hoc corrections were applied for multi‐group comparisons of protein levels. All values are presented as means ± SEM, n = 3–5 per group; *p < 0.05, **p < 0.01. CQ indicates chloroquine.

To further demonstrate the role of autophagy in Ang II‐induced AFs, we checked the effect of CQ, an autophagy antagonist, on Ang II treated AFs. As anticipated, CQ addition to Ang II treated cells further increased the protein levels of α‐SMA and Collagen I in AFs in contrast to Ang II alone treatment (Figure 5A,B). Consistent with the phenotypic transformation, the AFs treated with CQ and Ang II showed a higher level of proliferation compared to AFs treated with Ang II alone, as seen in the EdU assay (Figure 5C,D). Moreover, an increase in cell migration was seen in AFs treated by a combination of CQ and Ang II (Figure 5E,F). Furthermore, the upregulation of SQSTM1 (p62) and downregulation of LC3B‐II/I proved that Ang II restrained autophagic activity in AFs (Figure 5G,H). Taken together, we can conclude that autophagy is certainly involved in regulation of Ang II‐induced adventitial remodelling.

### 3.6 PI3K/AKT/mTOR signalling is required for DDR2‐mediated autophagy inhibition

**FIGURE 6:**
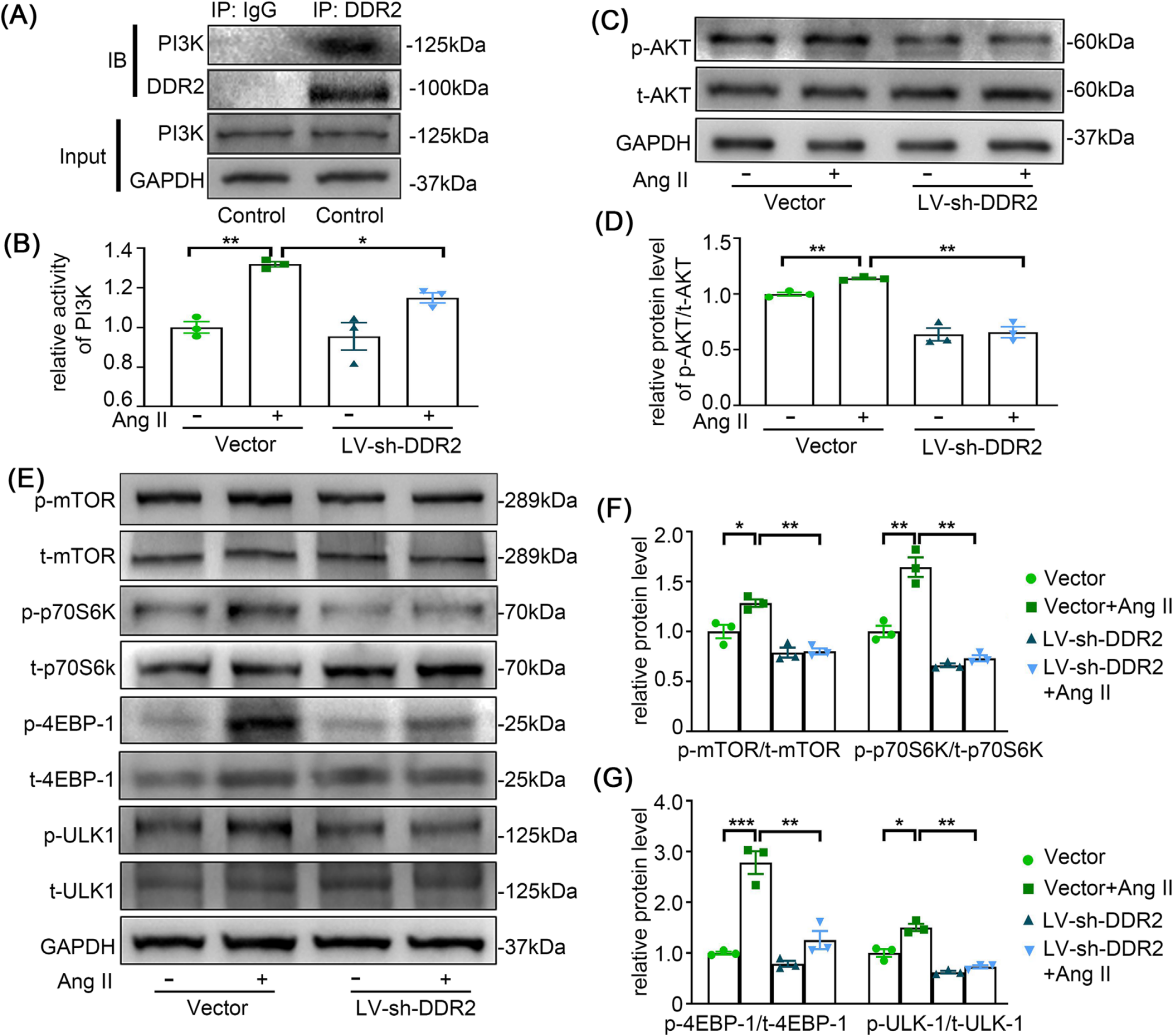
PI3K/AKT/mTOR signalling is required for discoidin domain receptor 2 (DDR2)‐mediated autophagy inhibition. AFs were infected with LV‐sh‐DDR2 or scramble for 24 h, followed by treatment with Ang II (100 nmol/L) for 24 h.(A) Representative Western blot of PI3K level following immunoprecipitation with anti‐DDR2 antibody in untreated AFs. (B) Quantification of the activity of PI3K by PI3K activity assay kit. (C‐D) The expression of p‐AKT and t‐AKT were examined by Western blotting and quantified by densitometry. (E‐G) The expression of p‐mTOR, t‐mTOR, p‐p70S6K, t‐p70S6K, p‐4EBP‐1, t‐4EBP‐1, p‐ULK1 and t‐ULK1 were examined by Western blotting and quantified by densitometry. Non‐parametric Kruskal–Wallis tests with Dunn's post hoc comparisons adjusted by the Benjamini‐Hochberg method were applied for multi‐group analyses. All values are presented as means ± SEM, n = 3–5 per group; *p < 0.05, **p < 0.01, ***p < 0.001.

Although we confirmed that DDR2 contributes to Ang II‐induced adventitial remodelling in association with autophagy, the underlying mechanisms to these phenomena are still needed to be explored. Previous studies have suggested a role of PI3K/Akt/ mTOR pathway in hypoxia‐induced AFs proliferation.^23^ Also, DDR2 has been implicated in PI3K pathway in some malignancies.^24^ To delineate whether Ang II modulates DDR2 through AT1 or AT2 receptor signalling, we performed pharmacological antagonism experiments.^25^ Pretreatment with the AT1 receptor blocker losartan completely abolished Ang II‐induced DDR2 upregulation, whereas the AT2 receptor antagonist PD123319 showed no inhibitory effect (Figure S3). This confirmed AT1 receptor specificity in mediating Ang II‐DDR2 signalling. We then performed a co‐immunoprecipitation assay to further explore whether PI3K is a target of DDR2. We found that endogenous PI3K was co‐immunoprecipitated with DDR2 in AF lysates (Figure 6A). Significantly, PI3K activity was elevated in response to Ang II stimulation, accompanied by enhanced phosphorylation of AKT, mTOR, and downstream effectors including p70S6K, 4E‐BP1, and ULK1, thereby inhibiting autophagy. However, the activation of PI3K/AKT/mTOR signalling was neutralized by the inhibition of DDR2 (Figure 6B–G). These results indicated that DDR2 mediates Ang II‐induced autophagy inhibition via PI3K/Akt/mTOR pathway.

### 3.7 PI3K/AKT signal inhibition attenuates Ang II‐induced adventitial remodelling

**FIGURE 7:**
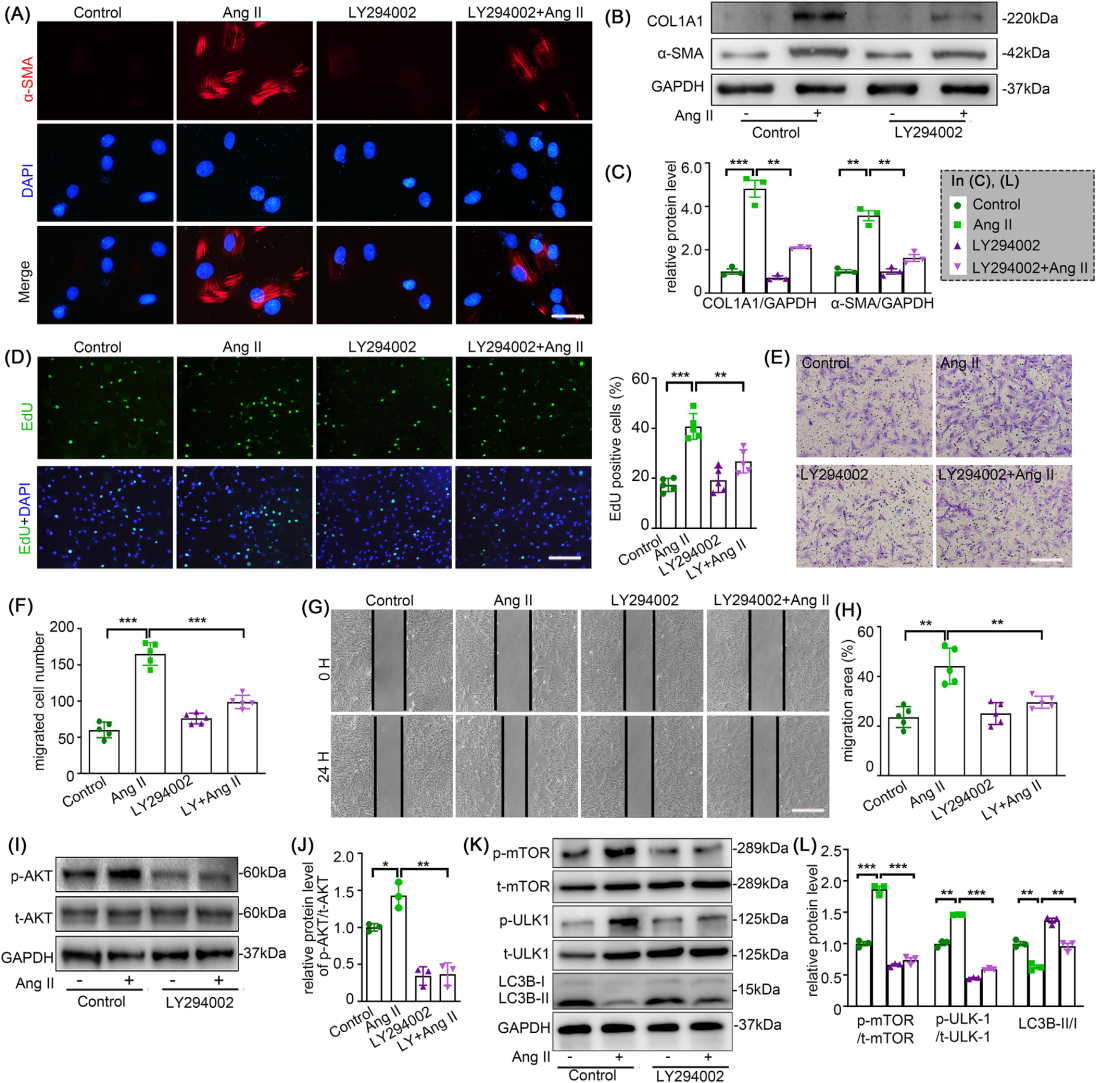
PI3K/AKT signal inhibition attenuated Ang II‐induced adventitial remodelling. Mouse AFs with or without LY294002 (20 εmM) pretreatment for 1 h were treated with either the vehicle or Ang II (100 nM) for 24 h. (A) AF phenotype switching was observed by immunofluorescence staining with α‐SMA (red) and DAPI (blue). Scale bar = 40 µm. (B‐C) The protein expression of α‐SMA and COL1A1 were examined by Western blotting and quantified by densitometry. (D) Representative images and quantification of EdU staining. Scale bar = 200 µm. (E‐F) Representative images of transwell migration assay and quantification of the migrated cells. Scale bar = 200 µm. (G‐H) Representative images of the in vitro scratch‐wound assay and quantification of the migration area were presented. Scale bar = 500 µm. (I‐L) The expression of p‐AKT, t‐AKT, p‐mTOR, t‐mTOR, p‐ULK1, t‐ULK1 and LC3B were examined by Western blotting and quantified by densitometry. Statistical significance was assessed using two‐way ANOVA followed by Bonferroni test. Non‐parametric Kruskal–Wallis tests with Dunn–Benjamini–Hochberg post hoc corrections were applied for multi‐group comparisons of protein levels. All values are presented as means ± SEM, n = 3–5 per group; *p < 0.05, **p < 0.01, ***p < 0.001. LY indicates LY294002.

LY294002, an inhibitor of the PI3K/AKT pathway, was used to further elucidate the importance of this pathway in the phenotypic alteration of AFs induced by Ang II. The elevated expression of α‐SMA and Collagen I by Ang II, along with intensive proliferation and migration and PI3K/Akt/mTOR pathway, were markedly attenuated after LY294002 intervention (Figure 7). These results further validated our hypothesis.

### 3.8 DDR2‐mediated autophagy inhibition promotes ROS accumulation in Ang II‐stimulated AFs

**FIGURE 8:**
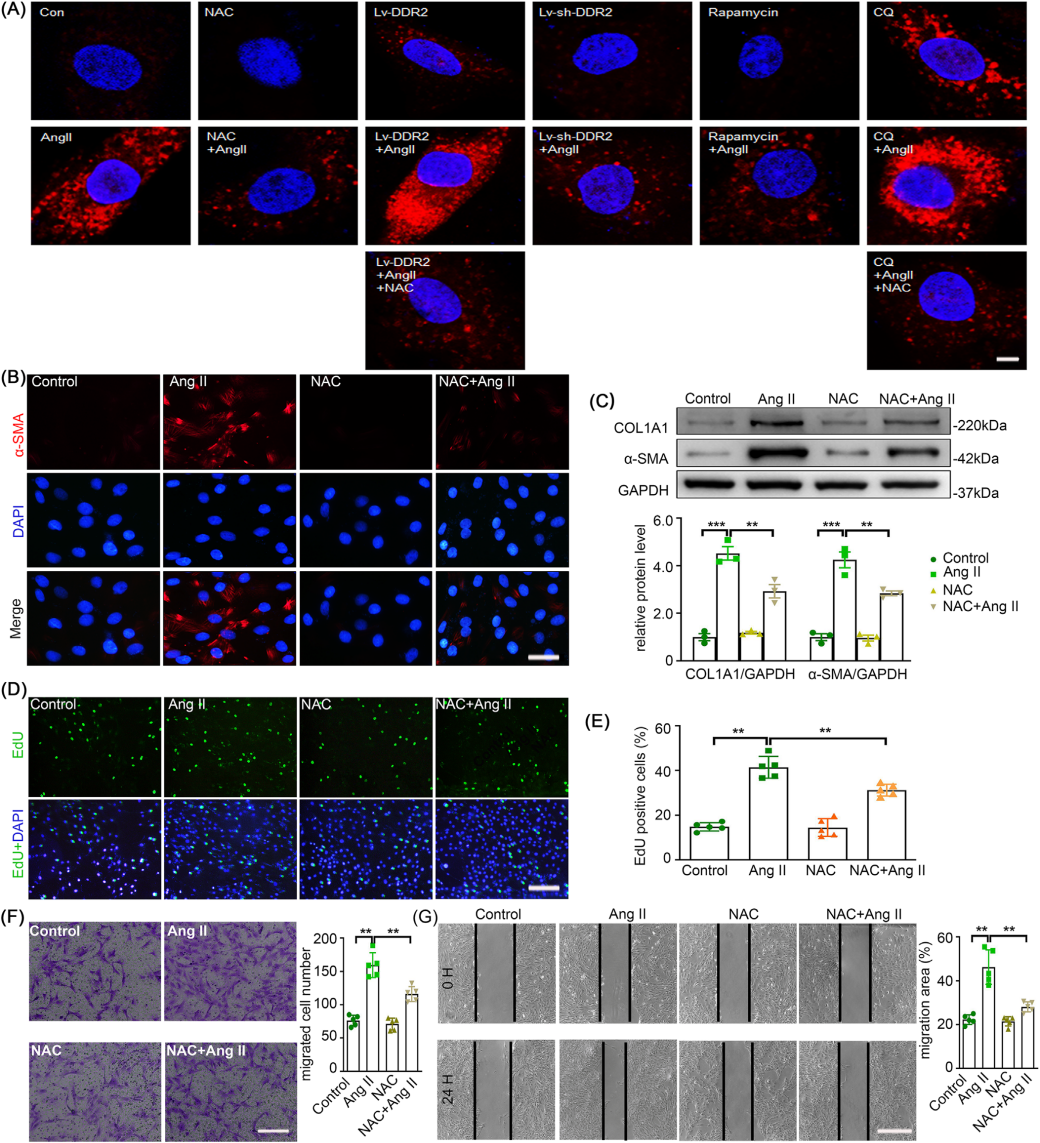
Discoidin domain receptor 2 (DDR2)‐mediated autophagy inhibition promotes ROS accumulation in Ang II stimulated AF. Mouse AFs with or without NAC (100 µmol/L) pretreatment for 1 h were treated with either the vehicle or Ang II (100 nmol/L) for 24 h. (A) Representative images of mitochondrial superoxide stained by MitoSOX. Scale bar = 40 µm. (B) AF phenotype switching was observed by immunofluorescence staining with α‐SMA (red) and DAPI (blue). Scale bar = 40 µm. (C) The protein expression of α‐SMA and COL1A1 were examined by Western blotting and quantified by densitometry. (D‐E) Representative images and quantification of EdU staining. Scale bar = 200 µm. (F) Representative images of transwell migration assay and quantification of the migrated cells. Scale bar = 200 µm. (G) Representative images of the in vitro scratch‐wound assay and quantification of the migration area were presented. Scale bar = 500 µm. Statistical significance was assessed using two‐way ANOVA followed by Bonferroni test. All values are presented as means ± SEM, n = 3–5 per group; **p < 0.01, ***p < 0.001.

In our recent study, we found that Ang II stimulation leads to massive ROS production partially contributing to the phenotypic switch of AFs. As autophagy is reported to be a tool for the balancing the amount of ROS in a cell, we investigated whether Ang II‐induced AFs phenotypic switching through inhibition of autophagy is related to redox homeostasis. We thus examined the ROS level of AFs upon Ang II stimulation and other treatments using MitoSOX staining. Notably, rapamycin and LV‐sh‐DDR2 both attenuated Ang II‐induced ROS elevation to a level comparable to the well‐known ROS scavenger NAC. Conversely, CQ and LV‐DDR2 further potentiated ROS elevation (Figure 8A). In addition, NAC significantly attenuated Ang II‐induced AFs phenotypic switching, cell proliferation and migration (Figure 8B–G). This proved that there is a role of autophagy in maintaining redox homeostasis during Ang II‐induced AFs activation.

### 3.9 Rapamycin partially attenuates Ang II‐induced adventitial remodelling and the phenotypic transition of AFs in C57BL/6J mice

**FIGURE 9:**
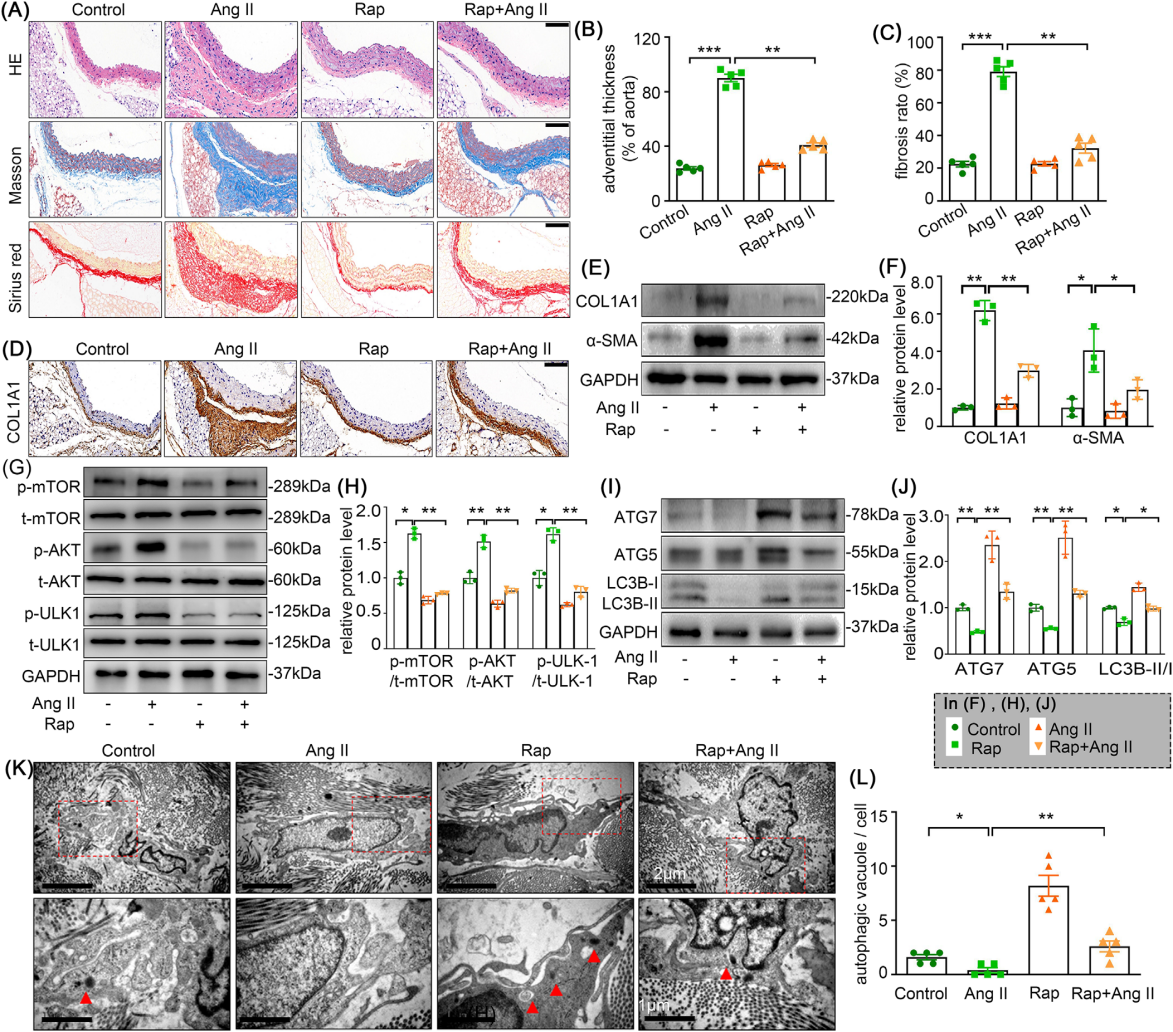
Rapamycin partially attenuated Ang II‐induced adventitial remodelling and AF phenotype switching in C57BL/6J mice. Saline or Ang II was administrated to mice for 4 weeks with or without rapamycin administration (4 mg/kg per day). (A) Representative haematoxylin and eosin (HE), Masson's trichrome and sirius red staining of the aortic cross‐sections. Scale bar = 100 µm. (B) Quantification of the adventitial thickness. (C) Quantification of the fibrosis ratio. (D) Representative collagen I staining of the aortic cross‐sections. Scale bar = 100 µm. (E‐F) The adventitial fibrosis indicated by the expression level of α‐SMA and COL1A1 were examined by Western blotting and quantified by densitometry; (G‐J) The expression of p‐mTOR, t‐mTOR, p‐AKT, t‐AKT, p‐ULK1, t‐ULK1, ATG7, ATG5 and LC3B were examined by Western blotting and quantified by densitometry. (K‐L) Electron micrograph of autophagosome (marked with arrow) from the adventitia of the respective group and the quantification of autophagic vacuole. Scale bar = 2 µm (upper) and 1 µm (down). Two‐way analysis of variance followed by the Bonferroni's post hoc test was used for statistical analyses. Non‐parametric Kruskal–Wallis tests with Dunn–Benjamini–Hochberg post hoc corrections were applied for multi‐group comparisons of protein levels. All values are presented as means ± SEM, n = 3–5 per group; *p < 0.05, **p < 0.01, ***p < 0.001.

To further elucidate whether autophagy is involved in adventitial remodelling, we induced adventitial remodelling in a C57BL/6J mice model infused with Ang II for 4 weeks. The H&E, Masson and Sirius red staining were performed to assess the aorta remodelling. As shown in the Figure 9A–C, rapamycin significantly reduced Ang II infusion‐induced adventitia thickening and fibrosis. Similarly, immunohistochemistry staining, and Western blot analysis also exhibited that rapamycin remarkably attenuated the Ang II‐induced collagen I and α‐SMA expression in mice, both of which are well‐recognized marker of AFs phenotype switching (Figure 9D–F). We next examined the changes of autophagy in Ang II infusion‐induced adventitia. Coinciding with our in vitro results, electron microscopy and Western blot analysis also showed that Ang II negatively regulates autophagy in adventitia, whereas rapamycin treatment can abolish this effect (Figure 9G–L). Notably, while rapamycin significantly attenuated Ang II‐induced adventitial remodelling and fibrosis, our quantitative analysis revealed incomplete restoration to baseline levels (Figure 9B,C, F, J, L; Figure S4). This partial therapeutic efficacy suggested that mTOR‐dependent autophagy modulation alone may be insufficient to fully counteract Ang II's pro‐fibrotic signalling, potentially due to parallel activation of mTOR‐independent pathways that sustain myofibroblast activation.^26^

### 3.10 Deletion of Ddr2 alleviates adventitial remodelling and autophagy restriction in mice with Ang II infusion

**FIGURE 10:**
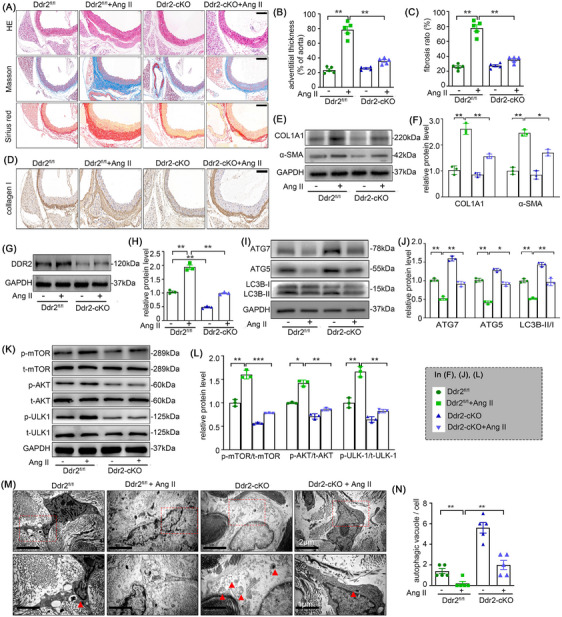
Deletion of Ddr2 alleviates adventitial remodelling and autophagy restriction in mice with Ang II infusion. Saline or Ang II was administrated to mice for 4 weeks in Ddr2‐cKO and Ddr2^fl/fl^ mice. (A) Representative haematoxylin and eosin (HE), Masson's trichrome and sirius red staining of the aortic cross‐sections. Scale bar = 100 µm. (B) Quantification of the adventitial thickness. (C) Quantification of the fibrosis ratio. (D) Representative collagen I staining of the aortic cross‐sections. Scale bar = 100 µm. (E‐F) The adventitial fibrosis indicated by the expression level of α‐SMA and collagen I were examined by Western blotting and quantified by densitometry; (G‐H) The protein expression of DDR2 was examined by Western blotting and quantified by densitometry; (I‐L) The expression of p‐mTOR, t‐mTOR, p‐AKT, t‐AKT, p‐ULK1, t‐ULK1, ATG7, ATG5 and LC3B were examined by Western blotting and quantified by densitometry. (M‐N) Electron micrograph of autophagosome (marked with arrow) from the adventitia of the respective group and the quantification of autophagic vacuole. Scale bar = 2 µm (upper) and 1 µm (down). Two‐way analysis of variance followed by the Bonferroni's post hoc test was used for statistical analyses. Non‐parametric Kruskal‐Wallis tests with Dunn‐Benjamini‐Hochberg post hoc corrections were applied for multi‐group comparisons of protein levels. All values are presented as means ± SEM, n = 3–5 per group; *p < 0.05, **p < 0.01, ***p < 0.001.

To clarify the role of DDR2 in adventitial remodelling and autophagy in vivo, we generated Ddr2‐cKO mice (Figure S5, Table 1) and exposed them to Ang II for 4 weeks. Deletion of Ddr2 obviously attenuated adventitia thickening and fibrosis in mice with Ang II infusion (Figure 10A–C). The Ang II‐induced collagen I and α‐SMA expressions in the mice adventitia were also sharply decreased, as confirmed by the immunohistochemistry staining and Western blot analysis (Figure 10D–F). Coinciding with our in vitro results, the expression of DDR2 was significantly increased after Ang II infusion in mice adventitia (Figure 10G,H). Electron microscopy and western blot analysis also demonstrated that the absence of DDR2 alleviated autophagy restriction in Ang II infusion‐induced adventitia (Figure 10I–N). Of note, DDR2 deficiency had negligible effect on the body weight and blood pressure (Figure S1A,B).

**TABLE 1:** Primer design for genotyping discoidin domain receptor 2 (DDR2) conditional knockout and Cre recombinase validation in mice.

Primer	Sequence 5' –> 3'	Primer type
**P1**	AGCTACCTCCCACCTGACTT	Forward
**P2**	TAGCATCCTGCTTCCTCCCA	Reverse
**P3**	ATGGGTCCCATTCCTCTGATCTCC	Forward
**P4**	CCATTTCCGGTTATTCAACTTGCAC	Reverse

Ddr2 allele‐specific primers (P1/P2), Amplifies both wild‐type (WT, 269 bp) and floxed Ddr2 alleles (331 bp) to distinguish genotypes. Cre recombinase validation primers (P3/P4): Detects Cre transgene expression (399 bp product). Positive control: Transgenic Cre mice (399bp), Negative control: Non‐transgenic mice with no amplification.

## 4 DISCUSSION

**FIGURE 11:**
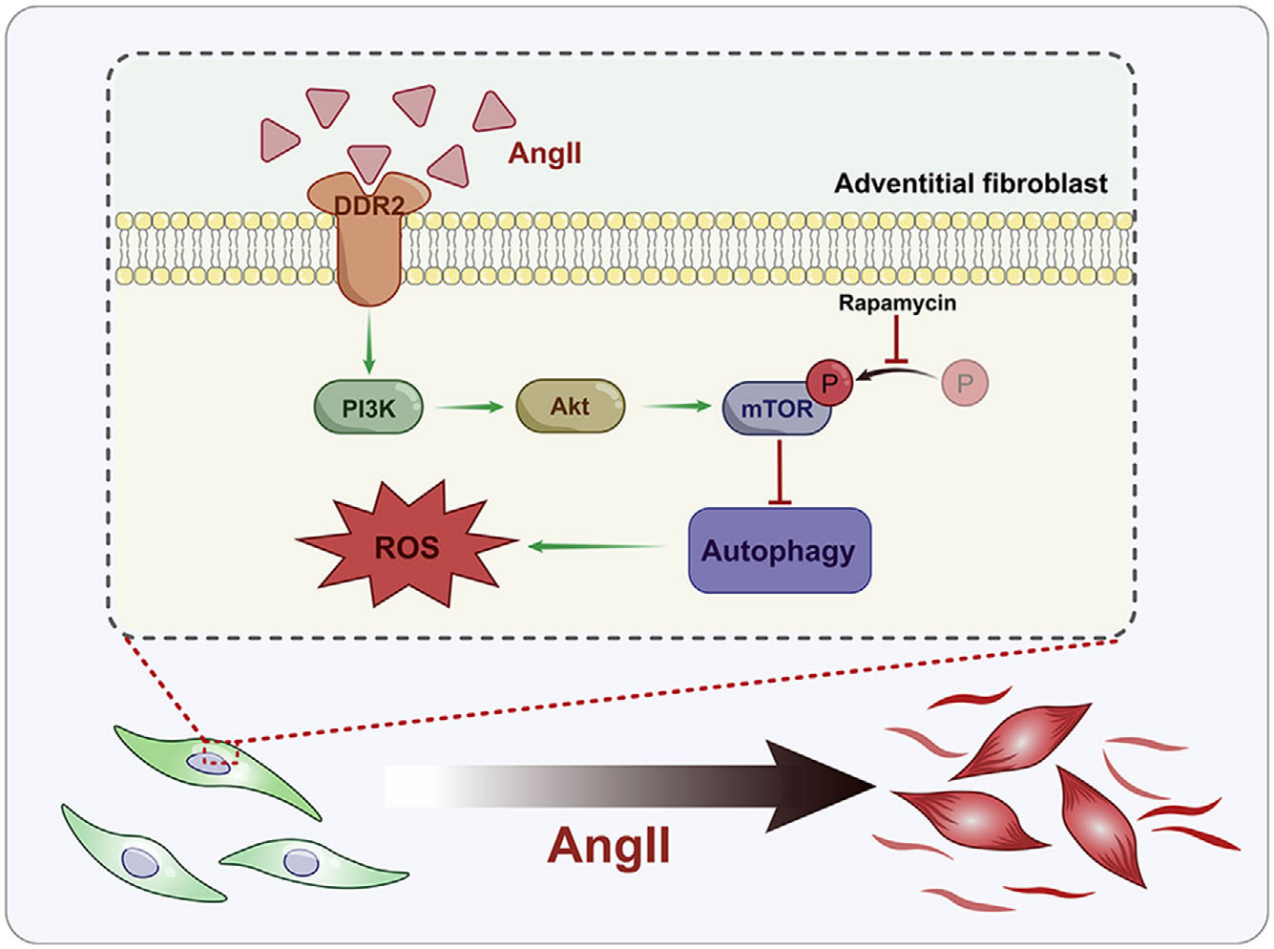
Discoidin domain receptor 2 (DDR2) promotes PI3K/Akt/mTOR mediated autophagy inhibition, which contributes to Ang II‐induced AF phenotypic switch.

Here, we demonstrated that DDR2 contributes to hypertensive phenotypic switch of AFs and adventitial remodelling by inhibiting autophagy and ROS clearance. Thus, this study revealed that DDR2 is a vital regulator of AF phenotypic switch and adventitial remodelling. Identifying the characteristics of DDR2 provides valuable mechanistic insight into the treatment of adventitial remodelling‐related diseases (See Figure 11)

Recently, AFs have been considered to play a vital role in vascular remodelling Ample evidence support that Ang II participates in AF phenotypic switching and adventitial remodelling in hypertension.^27^, ^28^ A growing number of studies have emerged, highlighting the role of collagen receptors in fibroblast activation during tissue restructuring.^29^, ^30^ DDR2, a collagen‐specific receptor, is reportedly involved in fibrotic diseases and plays a role in fundamental cellular events, such as extracellular matrix remodelling and phenotypic switching.^31^, ^32^ In line with these studies, we revealed that the mRNA expression of DDR2 in AF increased prominently in Ang II induced hypertensive mice. Interestingly, DDR2 has been implicated as a key regulator in cardiac fibrosis. George et al. revealed a positive feedback loop between DDR2 and collagen production.^33^ Since DDR‐sustained tyrosine phosphorylation is involved in the activation of PI3K/Akt/mTOR pathway,^34^, ^35^ which regulates autophagy in a wide range of cell types and is significantly activated in hypoxia‐induced AFs proliferation, we investigated the effects of DDR2 on Ang II induced AFs autophagy and phenotypic switch. Our results show that genetic inhibition of DDR2 significantly attenuated Ang II induced autophagy inhibition via the PI3K/Akt/mTOR pathway and inhibited AFs phenotypic switch. On the other hand, DDR2 overexpression markedly aggravated Ang II induced autophagy inhibition and AFs phenotypic switching. These effects that were effectively blocked by the DDR2‐specific inhibitor. Although the role of DDR2 in Ang II induced autophagy inhibition and AFs phenotypic switching is delineated, the underlying mechanism needs further study.

Our latest study demonstrated that Ang II facilitates a phenotypic shift in AFs by enhancing mitochondrial fission and subsequent ROS generation.^5^ Given that autophagy reportedly serves as a buffer system to control ROS levels,^36^ we investigated whether autophagy mediates Ang II‐induced AFs phenotypic switching and adventitial remodelling. We found that Ang II significantly inhibits autophagy and induces AFs phenotypic switching, migration and proliferation in vitro. These effects were partially attenuated by rapamycin (an autophagy activator) but exacerbated by CQ (an autophagy inhibitor). Similar results were observed in Ang II–induced remodelling aortas. These results implicate that autophagy may play a critical role in adventitial remodelling associated with ROS hyperactivation.

It's widely recognized that ROS positively influences the activation of autophagy in diverse stimulating conditions, contributing to redox balance maintenance.^37^ Conversely, defective autophagy (e.g., ATG7 deficiency) elevates intracellular ROS.^38^, ^39^ As expected, our results show that autophagy activation by rapamycin markedly attenuated Ang II‐induced ROS elevation, while autophagy inhibition by CQ significantly aggravated ROS elevation. In addition, ROS scavenger NAC was found to alleviated Ang II induced ROS and AFs phenotypic switching. These results further confirmed that Ang II induces AFs phenotypic switching through inhibition of autophagy related redox homeostasis. To our knowledge, this is the inaugural study to delineate the critical role of autophagy in the phenotypic switching of AFs, although its vital role in AFs migration and inflammation has been reported previously. Our research concentrated on a novel DDR2‐dependent mechanism by which Ang II‐suppresses autophagy and drives adventitial remodelling through the PI3K/Akt/mTOR signalling pathway in AFs. The participation of the PI3K‐Akt and mTOR signalling pathways in Ang II‐mediated autophagy impairment is evident across a range of cell types, encompassing both vascular endothelial cells and smooth muscle cells.^40^, ^41^ Furthermore, hypoxia‐induced proliferation in AFs is known to necessitate the activation and interplay of PI3K, Akt, and mTOR.^22^, ^23^ Nonetheless, prior to this study, no direct evidence linked this pathway to Ang II‐induced phenotypic alterations and adventitial remodelling in AFs. Crucially, we identified that Ang II activates DDR2 in an AT1 receptor‐specific manner, which subsequently recruits and activates PI3K via direct physical interaction. The formation of DDR2‐PI3K complex triggers a signalling cascade characterized by sequential phosphorylation of Akt, mTOR, and downstream effectors (p70S6K, 4E‐BP1, ULK1), ultimately suppressing autophagic flux.

Our study found that Ang II‐induced AFs phenotypic switching is significantly sensitive to PI3K inhibitor LY294002, indicating a critical role of PI3K signalling component in this response. Cooperating with the effect of Ang II on PI3K activity, Akt phosphorylation also increased upon exposure of AFs to Ang II. Ample evidence shows that mTOR plays a central role in the regulation of autophagy and it is in turn regulated by PI3K/AKT.^42^ In our study, we observed that Ang II significantly increased phosphorylation of mTOR and its downstream targets p70S6K, 4E‐BP1 and ULK1. Concurrently, Ang II downregulates key autophagy effectors, including ATG5, ATG7, and LC3B‐II/I, which disrupts autophagosome elongation and maturation. This mechanism aligns with the canonical role of Class I PI3K in mTOR‐dependent autophagy inhibition.^43^ In contrast, CQ exacerbated autophagic flux stagnation by blocking autophagosome‐lysosome fusion, as demonstrated by elevated SQSTM1 and reduced LC3B‐II/I levels. Notably, the relationship between ULK1 and autophagy is still controversial. ULK1 is also found to induce autophagy.^44^ These results suggest that PI3K/Akt/ mTOR signalling may be a critical target of Ang II in mediating autophagy suppression and phenotypic switching in AFs.

In accordance, fibroblast conditional DDR2 knockout in mice markedly alleviated PI3K/Akt/mTOR pathway mediated autophagy inhibition and retarded AF phenotypic switch and adventitial remodelling. No alteration in SBP in fibroblast DDR2 deficiency mice compared with DDR2^fl/fl^ littermates were observed, indicating that the effects of DDR2 on adventitial remodelling are independent of blood pressure regulation. Our study is typically the first to reveal the functional importance of DDR2 in PI3K/Akt/mTOR pathway mediated autophagy, AFs phenotypic switching and adventitial remodelling. Of note, there is a limitation in this study that the partial rescue of LC3‐II levels by rapamycin implies that Ang II may engage additional pathways beyond mTOR to modulate autophagy, future studies combining DDR2 genetic or pharmacological targeting with mTOR inhibition will be essential to dissect these parallel mechanisms.

In summary, our study demonstrated that Ang II‐induced adventitial remodelling is associated with autophagy suppression in AFs both in vitro and in vivo. Activation of autophagy via rapamycin or fibroblast‐specific DDR2 knockout attenuated Ang II‐driven AFs proliferation, migration, and phenotypic switching, and suppressed pathological adventitial remodelling. Mechanistically, DDR2/PI3K/Akt/mTOR signalling pathway emerged as a critical regulator of autophagy dysfunction, which mediated ROS accumulation and AFs activation. Therefore, DDR2 specific inhibition in adventitial fibroblast may represent a novel therapeutic strategy for addressing adventitial remodelling‐related diseases. In addition, these results provide a new implication for the understanding of the mechanisms underlying DDR2 regulated autophagy.

However, we acknowledge some critical limitations. Our experimental design involves pre‐treatment prior to Ang II exposure, primarily demonstrating the prevention of fibroblast activation and autophagy dysfunction. To directly test phenotypic reversal, future studies should administer interventions post‐Ang II challenge. Moreover, our data suggested that DDR2 is not the sole contributor of adventitial fibrosis. Further research should also consider^45^ drug access to vascular adventitia and other cellular contributors to aortic fibrosis.

## AUTHOR CONTRIBUTIONS

*Study concept and design*: All authors. *Cellular/molecular experiments and mechanistic investigations*: Gaojian Huang, Yuhao Zhao, Xuelian Wang, Tong Zhu. Single‐cell data analysis: Yuhao Zhao. *Manuscript drafting*: Gaojian Huang. *Animal reproduction validation and experimental procedures*: Gaojian Huang, Zhilei Cong, Xuelian Wang, Yuhao Zhao. *Critical revision of the manuscript*: All authors. *Statistical analysis*: Xuelian Wang, Ruosen Yuan, Gaojian Huang.

## CONFLICT OF INTEREST STATEMENT

The authors declare no conflicts of interest.

## ETHICS STATEMENT

All animal experiments were conducted in compliance with the Guidelines on the Care and Use of Laboratory Animals (NIH Publication No.85‐23, revised 1996) and were reviewed and approved by the Institutional Animal Care and Use Committee (IACUC) of Shanghai Jiaotong University. This approval encompasses the original study design, all subsequent amendments, and every procedure involving animal handling throughout the research.

## Supporting information



Supporting Information

## Data Availability

All data associated with this study are present in the paper or the Supplementary Information. Raw and analyzed RNA‐sequencing data generated during this study, we have uploaded the raw scRNAseq data to the GEO database (GSE number: [GSE282397]).
