# Supplementary material for: DDR2‐mediated autophagy inhibition contributes to angiotensin II‐induced adventitial remodeling
Source: Clin Transl Med. 2025 Jun 4;15(6):e70361. doi: 10.1002/ctm2.70361 (PMC12137622; doi:10.1002/ctm2.70361)
Supplement: Supplementary file 1 — Supporting Information [file CTM2-15-e70361-s001.docx]

**Title:** DDR2-mediated Autophagy Inhibition Contributes to Angiotensin II-Induced Adventitial Remodeling

**Short title:** DDR2 Contributes to Adventitial Remodeling

Gaojian Huang^1,3*^, Zhilei Cong^5*^, Yuhao Zhao^2^, Tong Zhu^1^, Ruosen Yuan^4^, Zhen Li^2#^, Xuelian Wang^1,4#^, Jia Qi^1#^

# corresponding author

^1^Department of Clinical Pharmacy, Xinhua Hospital affiliated to Shanghai Jiaotong University School of Medicine, Shanghai, China

^2^Department of Geriatric, Xinhua Hospital affiliated to Shanghai Jiaotong University School of Medicine, Shanghai, China

^3^Department of Cardiology, Shanghai Ninth People’s Hospital affiliated to Shanghai Jiaotong University School of Medicine, Shanghai, China

^4^Department of Cardiology, Ruijin Hospital affiliated to Shanghai Jiaotong University School of Medicine, Shanghai, China

^5^Department of Emergency, Huashan Hospital affiliated to Fudan University, Shanghai, China.

*These authors contribute equally.

**Figure S1. Time-Dependent Upregulation of DDR2 Protein in AFs Upon Ang II Stimulation.**


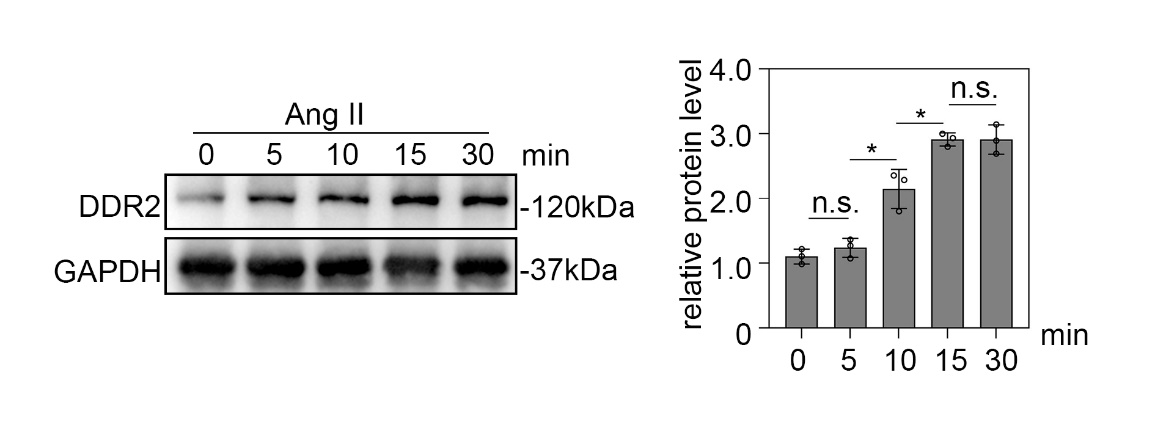


Representative western blots and quantification of DDR2 in AFs treated with Ang II (100 nmol/L) for indicated durations (0, 5, 10, 15, 30 min). The result show that Ang II induces acute activation of DDR2 protein within minutes.The data represent as mean ± SEM, n = 3. **p* < 0.05, *n.s.*: not significant. Non-parametric Kruskal-Wallis tests with Dunn's post hoc comparisons adjusted by the Benjamini-Hochberg method were applied.

**Figure S2. DDR2-Specific Inhibitor WRG-28 Blocks LV-DDR2-Enhanced Pro-Fibrotic Effects in AFs**


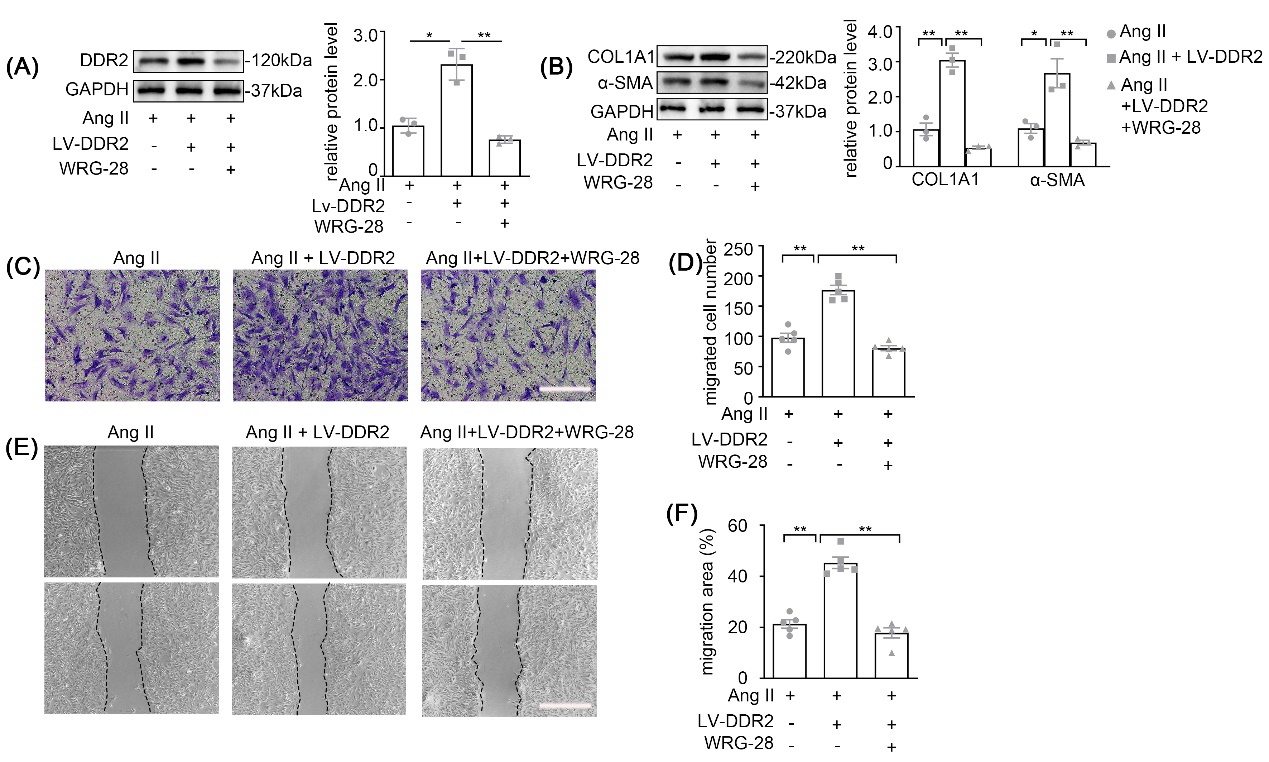


AFs were infected with LV-DDR2 for 24 h, with or without WRG-28 (1 μM) pretreatment for 1 h were treated followed by treatment with Ang Ⅱ (100 nM) for 24 h. (A-B) The expression of DDR2, COL1A1 and α-SMA were examined by Western blotting and quantified by densitometry. Non-parametric Kruskal-Wallis tests with Dunn's post hoc comparisons adjusted by the Benjamini-Hochberg method were applied for multi-group analyses. (C) Representative images of transwell migration assay and quantification of the migrated cells. Scale bar = 200 μm. (D) Representative images of scratch-wound assay and quantification of the migration area were presented. Scale bar = 500 μm. Two-way analysis of variance followed by the Bonferroni’s post hoc test was used for statistical analyses. All values are presented as means ± SEM, n = 3-5 per group; **p* < 0.05, ***p* < 0.01.

**Figure S3. DDR2 Overexpression Exacerbates Angiotensin II-Induced Fibrotic Responses in AFs via AT1 Receptor-Dependent Signaling**


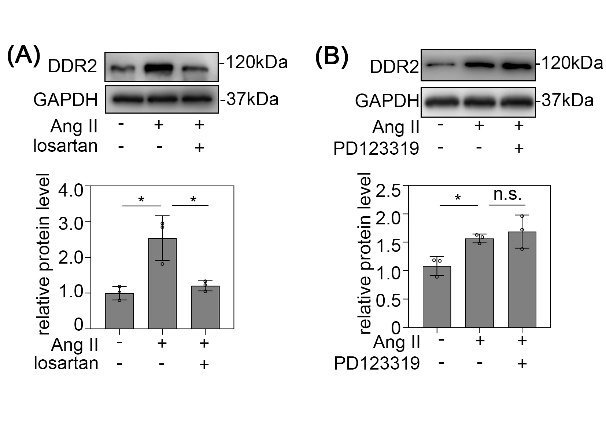


Pretreatments were performed by adding losartan (a selective AT1 receptor antagonist) or PD123319 (a selective AT2 receptor antagonist) before exposing cells to AngII. (A) Western blot analysis of DDR2 expression in AFs treated with Ang II (100 nM, 24 h) with or without losartan (1 μM). (B) Western blot and corresponding quantification of DDR2 expression under Ang II stimulation with or without PD123319 (1 μM). Data presented as mean ± SEM (n=3); statistical significance determined by Non-parametric Kruskal-Wallis tests with Dunn's post hoc comparisons adjusted by the Benjamini-Hochberg method. **p* < 0.05. *n.s.*: not significant.

**Figure S4. Statistical analysis supplement to Figure 9-Rapamycin Partially attenuates Ang Ⅱ-induced adventitial remodeling and the phenotypic transition of AFs in C57BL/6J mice**

**
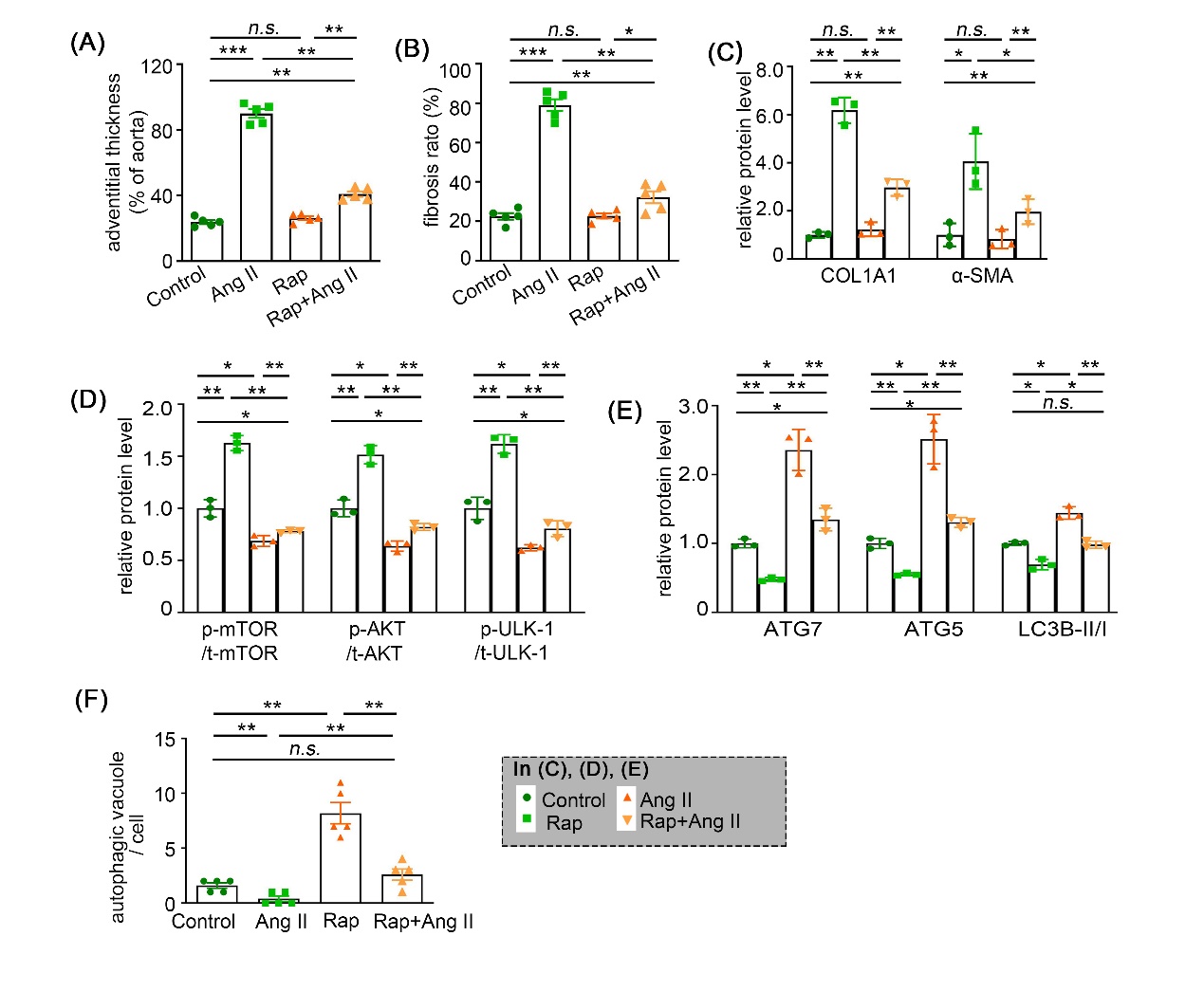
**

Statistical analysis supplement to Figure 9. (A) corresponds to 9B, (B) corresponds to 9C, (D) corresponds to 9F, € corresponds to 9J, and F corresponds to L. Rapamycin significantly attenuated Ang II-induced adventitial remodeling and fibrosis,while our quantitative analysis revealed incomplete restoration to baseline levels.

**Figure S5.** **Validation of Fibroblast-Specific DDR2 Conditional Knockout and Vascular Phenotypic Analysis in Mice**

**
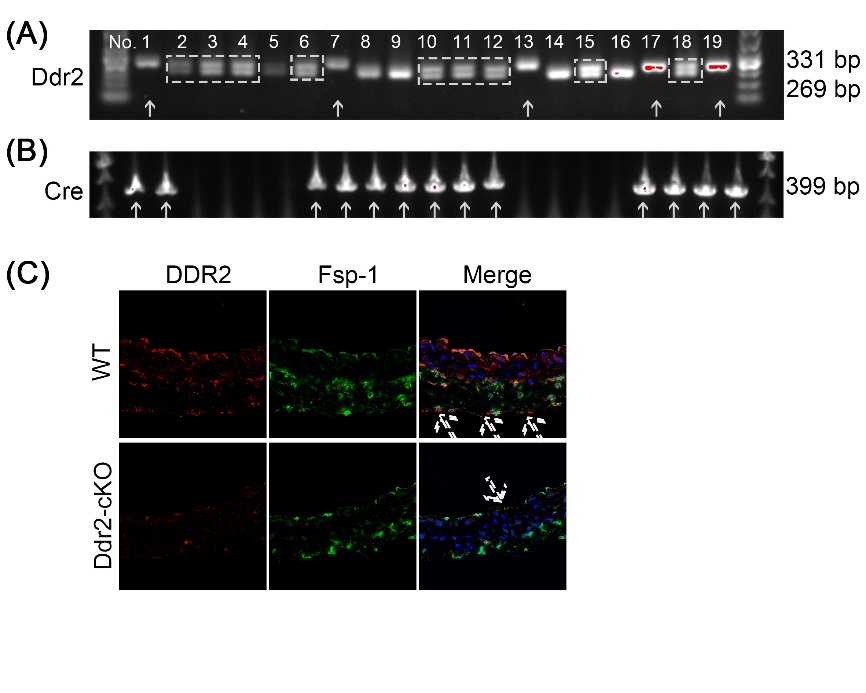
**

The Ddr2-floxed allele was detected using primers P1/P2 to assess the presence status of the floxed region, while the Cre recombinase transgene was identified through primers P3/P4 to confirm its presence (Table 1). (A) WT: one band with 269 bp, No. 5, 8, 9,14, 16; Heterozygous: two bands with 269 and 331 bp, No. 2, 3, 4, 10, 11, 12, 15,18; Homozygous: one band with 331 bp, No. 1,7,13,17,19. (B) 399 bp band，onfirmed presence of Cre transgene cassette; No band: Cre system inactivation. (C) Immunofluorescence Co-Staining of DDR2 and FSP-1. Fibroblast-specific marker co-localizes with DDR2 (yellow signal in merged image). Ddr2-cKO: diminished DDR2 co-localization.

**Figure S6. Effect of Ang II Stimulation on Body Weight and Blood Pressure in DDR2 Knockout Mice.**


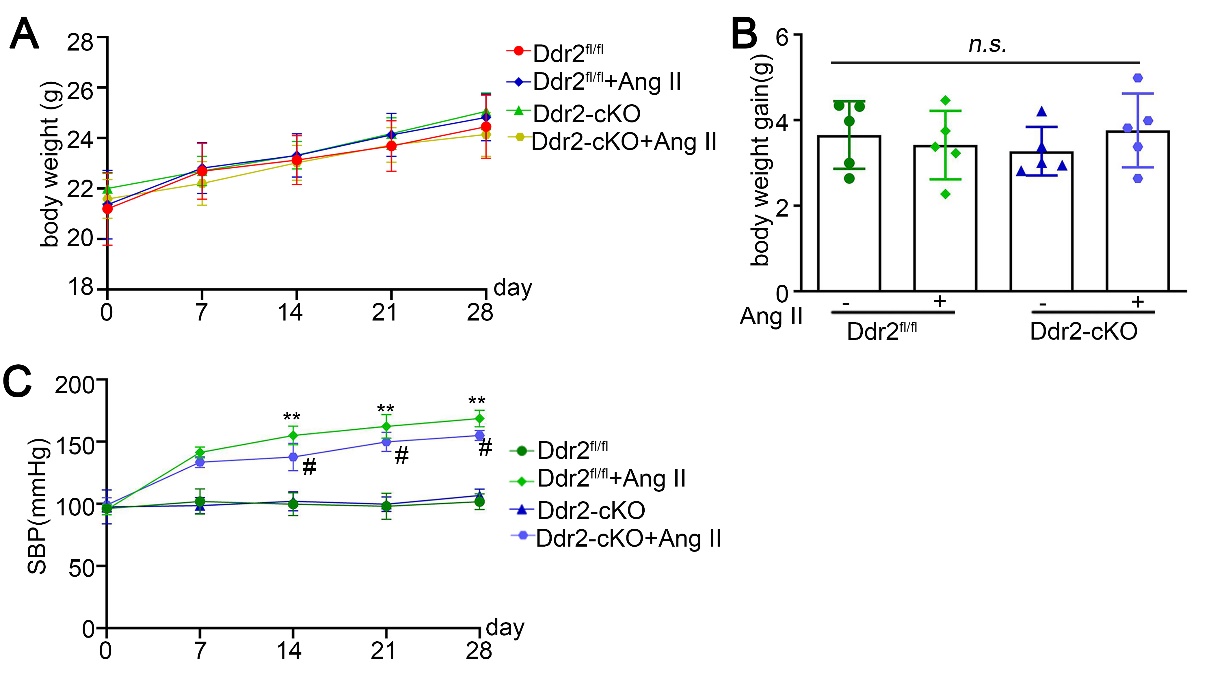


The figure shows the changes in body weight and blood pressure in Ddr2knockout (Ddr2-cKO) mice following Angiotensin II (Ang II, 1000 ng/min/kg) stimulation. (A-B) The body weight was monitored and quantified by weight gain in Ddr2-cKO mice and control mice following Angiotensin II (Ang II) infusion. Values are shown as means ± SEM (n = 5 mice per group). (C) Systolic blood pressure was measured weekly in both Ddr2-cKO and control mice after Ang II infusion. Values are shown as means ± SEM (n = 5 mice per group). ***p*<0.01, compared with DDR2^fl/fl^; *#p*<0.05 compared with DDR2^fl/fl^ + Ang II group. Two-way analysis of variance followed by the Bonferroni’s post hoc test was used for statistical analyses.
